# Characterization of differences in immune responses during bolus and continuous infusion endotoxin challenges using mathematical modelling

**DOI:** 10.1113/EP091552

**Published:** 2024-03-11

**Authors:** Kristen A. Windoloski, Susanne Janum, Ronan M. G. Berg, Mette S. Olufsen

**Affiliations:** ^1^ Department of Mathematics North Carolina State University Raleigh North Carolina USA; ^2^ Frederiksberg and Bispebjerg Hospitals Frederiksberg Denmark; ^3^ Department of Biomedical Sciences University of Copenhagen Copenhagen Denmark; ^4^ Department of Clinical Physiology and Nuclear Medicine and, Centre for Physical Activity Research Copenhagen University Hospital Copenhagen Denmark; ^5^ Neurovascular Research Laboratory University of South Wales Pontypridd UK

**Keywords:** administration method, continuous infusion, cytokines, data analysis, endotoxin challenge, inflammation, mathematical modelling

## Abstract

Endotoxin administration is commonly used to study the inflammatory response, and though traditionally given as a bolus injection, it can be administered as a continuous infusion over multiple hours. Several studies hypothesize that the latter better represents the prolonged and pronounced inflammation observed in conditions like sepsis. Yet very few experimental studies have administered endotoxin using both strategies, leaving significant gaps in determining the underlying mechanisms responsible for their differing immune responses. We used mathematical modelling to analyse cytokine data from two studies administering a 2 ng kg^−1^ dose of endotoxin, one as a bolus and the other as a continuous infusion over 4 h. Using our model, we simulated the dynamics of mean and subject‐specific cytokine responses as well as the response to long‐term endotoxin administration. Cytokine measurements revealed that the bolus injection led to significantly higher peaks for interleukin (IL)‐8, while IL‐10 reaches higher peaks during continuous administration. Moreover, the peak timing of all measured cytokines occurred later with continuous infusion. We identified three model parameters that significantly differed between the two administration methods. Monocyte activation of IL‐10 was greater during the continuous infusion, while tumour necrosis factor α and IL‐8 recovery rates were faster for the bolus injection. This suggests that a continuous infusion elicits a stronger, longer‐lasting systemic reaction through increased stimulation of monocyte anti‐inflammatory mediator production and decreased recovery of pro‐inflammatory catalysts. Furthermore, the continuous infusion model exhibited prolonged inflammation with recurrent peaks resolving within 2 days during long‐term (20–32 h) endotoxin administration.

## Abstract (highlights)

- **What is the central question of this study?**
  How do dynamic immune responses to continuous and bolus endotoxin administration compare using mathematical models, and what physiological mechanisms do we hypothesize attribute to these observed differences? How do extended and enhanced continuous infusions impact immune responses, and do they express similar characteristics to infection dynamics?
- **What is the main finding and its importance?**
  This study proposes a mathematical model for a continuous infusion of endotoxin. We hypothesize that the dynamic switch from a bolus to a continuous infusion of endotoxin, which results in higher peak concentrations of IL‐10, lower peak concentrations of IL‐8, and later peak timing of all measured cytokines, is attributed to increased monocyte activation rate of anti‐inflammatory cytokine IL‐10 and decreased decay rates of pro‐inflammatory cytokines TNF‐a and IL‐8. Additionally, prolonged LPS administration significantly extends the cytokine response and results in recurrent cytokine spikes. Our results provide a better understanding of the mechanisms impacting the change in immune responses with long‐term endotoxin infusion.

## 1 INTRODUCTION

Endotoxin (lipopolysaccharide, LPS), derived from the outer membrane of gram‐negative bacteria (Heine et al., 2001), is an immunostimulant administered to healthy subjects as an experimental procedure to study the inflammatory response (Suffredini & Noveck, 2014). This type of experiment, referred to as an endotoxin challenge, has allowed insight into mechanisms and treatments of inflammation events such as rheumatoid arthritis (Lorenz et al., 2013; Merrill et al., 2011; M. Miller et al., 1979), systemic lupus erythematosus (Zuckerman et al., 1996), cancer (Easson et al., 1998; Ho et al., 2013; Yassine, 2016), Alzheimer's disease (Akimoto et al., 2007; Cunningham et al., 2005; Sly et al., 2001) and sepsis (Fitzal et al., 2003; Fredriksson et al., 2009; Leijte et al., 2019; Shinozaki et al., 2010).

In an endotoxin challenge, LPS can be administered as a bolus (instantaneous) injection (Clodi et al., 2008; Copeland et al., 2005; Janum et al., 2016), a continuous infusion over several hours (Berg et al., 2012) or a combination of the two (Kiers et al., 2017) in both humans and animals (Bahador & Cross, 2007). The response is an increase in pro‐ (tumour necrosis factor α (TNF‐α), interleukin (IL) (IL‐1β, IL‐6, IL‐8) and anti‐ (IL‐10, IL‐1R α) inflammatory cytokines, immune cells, body temperature, heart rate, blood pressure and hormone levels (Bahador & Cross, 2007; Clodi et al., 2008; Givalois et al., 1994; Janum et al., 2016). The peak of each measured quantity and the time it takes to reach the peak vary depending on a host of controllable (administration method and total dose administered) and uncontrollable (individual variation due to genetics, sex and health status) factors.

Taudorf et al. (2007) performed an endotoxin challenge in healthy men, administering 0.3 ng kg^−1^ of LPS as a bolus and a continuous infusion over 4 h. They found that the administration method significantly affects TNF‐α, IL‐6 and neutrophil production rates. These quantities peaked earlier and had larger magnitudes during the bolus administration than the continuous infusion. Kiers et al. (2017) compared immune responses to 1 and 2 ng kg^−1^ LPS bolus doses to a 1 ng kg^−1^ bolus dose followed by a 3 ng kg^−1^ continuous infusion over 3 h. Results showed significant differences in mean cytokine concentrations, flu‐like symptoms (headache, nausea, shivering, pain), temperature and heart rate increases between the bolus‐only and the bolus plus continuous infusion. Cytokine responses (TNF‐α, IL‐6, IL‐8, IL‐10) reached significantly higher peak levels, and subjects exhibited prolonged elevated flu‐like symptoms during the bolus plus continuous infusion method. These results demonstrate that continuous infusion initiates a more durable and occasionally more pronounced impact on the immune response during the incitement of inflammation.

Although experimental studies are suitable for investigating the effects of the endotoxin administration method, they provide little insight into why differing dynamics are observed. This is where the power of mathematical modelling of physiological systems can be applied. Simulations with mathematical models can highlight the underlying mechanisms of disease, aid in disease diagnosis, test and validate treatments and predict patient trajectory and mortality. Numerous mathematical models of inflammation have been developed over the last two decades. Kumar et al. (2004), Day et al. (2006) and Reynolds et al. (2006) developed small but novel mathematical models, highlighting their ability to reproduce inflammation scenarios of clinical relevance and potential to predict treatment strategies. Several models built upon this foundation by adding specific immune cells and cytokines activated during the inflammatory response (Brady et al., 2018; Chow et al., 2005; Foteinou et al., 2009; Parker et al., 2016; Roy et al., 2007; Su et al., 2009; M. Torres et al., 2019). Others created detailed models incorporating feedback from other physiological entities such as the cardiovascular system, nervous system, the hypothalamic–pituitary–adrenal (HPA) axis, pain perception and thermal responses (Bangsgaard et al., 2017; Dobreva et al., 2021; Foteinou et al., 2011; Malek et al., 2015; Scheff et al., 2010; Windoloski et al., 2023). These studies demonstrate the need for computational inflammation models that (i) utilize experimental data from a continuous infusion of endotoxin and (ii) investigate the mechanisms behind response differences observed during variations in the endotoxin administration method.

Recent experimental studies (Kiers et al., 2017; van Lier et al., 2019) propose that a continuous endotoxin infusion is more appropriate to study the prolonged system response during systemic inflammation and sepsis. To provide more insight into understanding which immune signalling components are impacted during the switch from a bolus to continuous infusion, we studied the inflammatory response to continuous infusion of endotoxin through the lens of a mathematical model. Doing so provides (i) newfound insight into the response differences between a bolus and continuous administration of endotoxin, (ii) a better mathematical representation to study the dynamics of sepsis, and (iii) a better model to investigate treatments of inflammatory conditions since the continuous infusion prolongs the exposure window for treatment testing.

We present a novel inflammatory response mathematical model predicting innate cytokine responses (TNF‐α, IL‐6, IL‐8, IL‐10) to a 2 ng kg^−1^ bolus and 4‐h continuous infusion of endotoxin calibrated to experimental data from Berg et al. (2012) and Janum et al. (2016). The model structure is rigorously explored through sensitivity and identifiability analysis, and parameter estimation calibrates the model to mean and subject‐specific cytokine data. We compared each study's cytokine data to characterize larger endotoxin doses than compared in previous literature and developed statistical uncertainty bounds for the optimal mean model. This hypothesis‐generating study suggests mechanisms responsible for immune dynamics in bolus and continuous infusion experimental studies via statistical analysis of optimized model parameters. We propose that transitioning from a bolus to continuous infusion impacts IL‐10 activation by monocytes and TNF‐α and IL‐8 degradation rates. Moreover, we used our continuous infusion model to investigate the system response to perturbations in infusion duration and total endotoxin dose administered. This illustrates its capability as a clinically realistic in silico model that can simulate prolonged and pronounced responses.

## 2 METHODS

### 2.1 Ethical approval

The current work utilized experimental data from two published studies by Berg et al. (2012) and Janum et al. (2016). The study by Berg et al. (2012) was approved by the Scientific Ethical Committee of Copenhagen and Frederiksberg Municipalities in Denmark. The data from Berg et al. (2012) were made available by Berg (coauthor). The study by Janum et al. (2016) received approval for the experimental protocol by the Regional Committee on Health Research Ethics and the Regional Monitoring Board, and the study followed the protocols listed in the *Declaration of Helsinki*. Individual data from Janum et al. (2016) were made available by Janum (coauthor) and Mehlsen (coauthor of Janum et al., 2016). All participants from both studies gave their written and oral consent.

### 2.2 Experimental data

The experiments by Berg et al. (2012) and Janum et al. (2016) administered the same total dose of endotoxin (2 ng kg^−1^) to healthy study participants, one as a continuous infusion and one as a bolus injection. Mean and subject‐specific cytokine data were obtained and used to calibrate our mathematical model.

Berg et al. (2012) investigated the effects of an increase in mean arterial pressure on cerebral autoregulation. This study included nine healthy male participants aged 21–25. All study participants were subject to physical examination. Data were only included from subjects with normal blood work and cardiovascular markers. Participants did not take medication, had a typical medical history, were non‐sedentary and were infection‐free at least 4 weeks before the study. The study by Janum et al. (2016) was designed to investigate the connection between pain and the innate immune system reaction in 20 male athletes aged 18–35. All study participants had a healthy weight, were non‐smokers and had no signs of illness 2 weeks before the study day. Pre‐screening activities involved a review of each subject's medical history, a physical examination and laboratory work.

**FIGURE 1:**
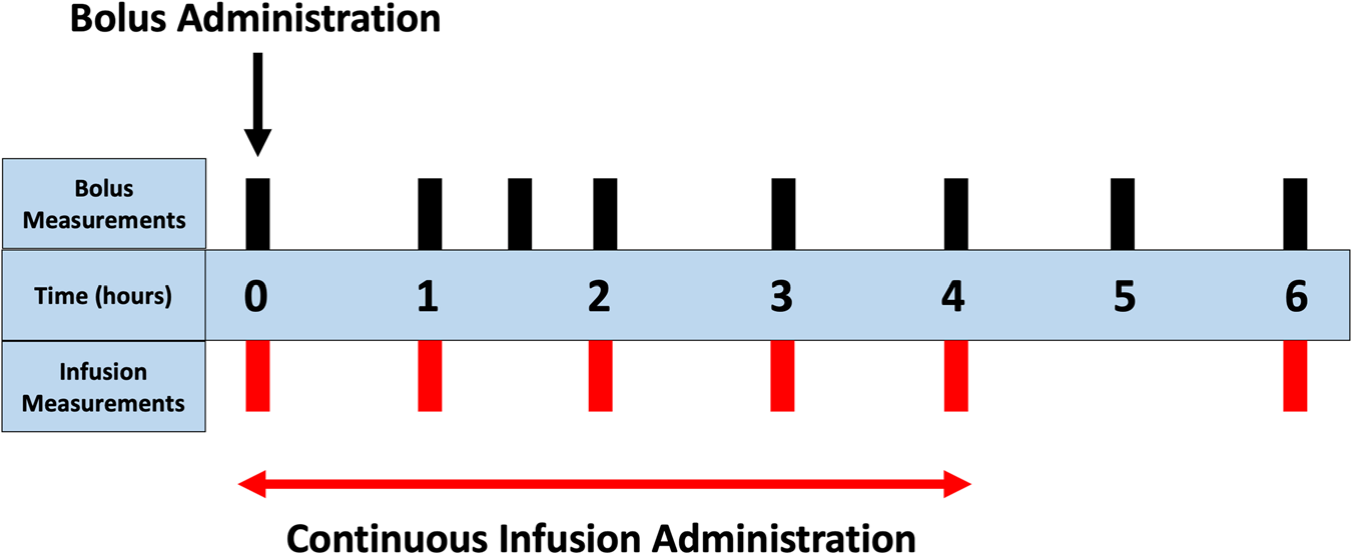
Administration times for the bolus (black arrow) (Janum et al., 2016) and continuous infusion (red arrow) (Berg et al., 2012). The times that measurements were taken for each study are denoted by the black (bolus) and red (continuous infusion) bars.

In Berg et al. (2012), participants were subject to a 4‐h continuous infusion of 2 ng kg^−1^ (0.5 ng kg^−1^ h^−1^) of endotoxin (Batch G2 B274, US Pharmacopeial Convention, Rockville, MD, USA) administered via an antecubital catheter. In contrast, the study participants in Janum et al. (2016) received a 2 ng kg^−1^ bolus endotoxin dose (Lot EC‐6, National Institutes of Health, Bethesda, MD, USA) via a peripheral intravenous catheter following 2 h of baseline stabilization. In Berg et al. (2012), blood samples were taken hourly for the first 4 h following the start of endotoxin administration and 2 h after completed endotoxin administration. In Janum et al. (2016) measurements were taken hourly, starting 2 h before endotoxin administration and continuing for 6 h after administration. An additional blood sample was taken 1.5 h after LPS administration to capture the peak response. Endotoxin administration and cytokine measurement times for each study are shown in Figure 1. Janum et al. (2016) used ELISA (Meso Scale Discovery, Rockville, MD, USA) and Berg et al. (2012) used SECTOR Imager 2400 (Meso Scale Diagnostics, Rockville, MD, USA) to determine concentrations of TNF‐α, IL‐6, IL‐8 and IL‐10.

**FIGURE 2:**
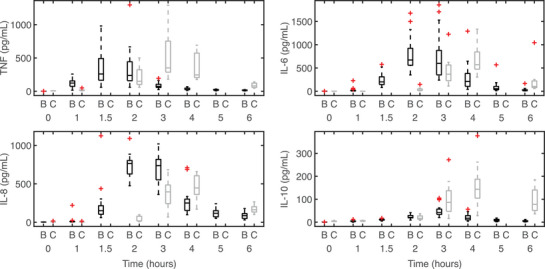
Box and whisker plots of the continuous infusion (m=9 subjects) (Berg et al., 2012) and bolus (n=20 subjects) (Janum et al., 2016) data. Black boxplots above the symbol ‘B’ represent bolus data, and grey boxplots above the symbol ‘C’ represent continuous infusion data. The red cross symbol denotes abnormal responses (outliers) from each study. This figure was generated using MATLAB code adapted from Danz (2023).

Subjects 1, 2, 6, 8 and 9 from the continuous infusion study were missing one cytokine measurement, and subject 4 was missing four measurements. Of these, subjects 4, 8 and 9 were missing baseline concentration measurements of IL‐6. Subject 4 also did not have a baseline concentration of IL‐10. Figure 2 shows data from both studies, identifying outliers from both data sets. Because the immune response exhibits significant variation in individual responses to stimuli, we considered these outlying data points abnormal but not unrealistic.

Because of the small sample sizes, the influence of abnormal data points on the average cytokine response is significant. Therefore, we calculated the mean cytokine response after removing outlying data points outside 1.5 × interquartile range (IQR), where the IQR encompasses the 25th and 75th percentile of data for each endotoxin data set. Because of the small number of study participants, we only removed abnormal (outlying) measurements instead of that individual's entire cytokine profile. Since our data were approximately normally distributed, the whisker length was set at MATLAB's default (1.5 × IQR). We used the mean of the bolus and continuous infusion data to calibrate our mathematical model. In the remainder of this study, we refer to this as the mean bolus or continuous endotoxin administration.

Mean and subject‐specific cytokine characteristics are reported in Table 1. Primary pro‐ and anti‐inflammatory cytokines TNF‐α and IL‐10 had higher mean peak concentrations during continuous infusion, while the secondary cytokine IL‐6 did not depend on the administration method, and IL‐8 had a higher peak value for bolus injection. With the continuous infusion, peak concentrations were later for all measured cytokines. Individual subject concentrations from both studies displayed a considerable variation in cytokine responses, where TNF‐α, IL‐6 and IL‐8 had a higher variation with bolus injection. Cytokine baseline values reported in Table 1 are all within the normal range (Biancotto et al., 2013), but the continuous infusion study has higher baseline values for TNF‐α and IL‐10 than the bolus study. We hypothesize that this variation could stem from (i) natural differences in cytokine levels in healthy individuals, which are known to vary markedly (Taudorf et al., 2008), (ii) different study protocols between the two groups, such as the impact of invasive procedures like catheterization on physiological stress, or (iii) different experimental time courses of the studies, which may have given rise to systematic error in the assaying method.

**TABLE 1:** Experimental data characteristics from the bolus and continuous infusion data provided in Janum et al. (2016) and Berg et al. (2012), respectively. The mean baseline value, peak value, and peak timing value (excluding outlying or missing data points) and the range of subject‐specific peak values and peak timing values are given.

Study	TNF‐α	IL‐6	IL‐8	IL‐10
Mean (SD) baseline concentration (pg ml^−1^)				
Bolus Continuous	1.02 (0.155) 6.24 (1.38)	0.911 (0.314) 0.610 (0.159)	3.01 (0.767) 2.96 (0.648)	0.204 (0.0864) 4.20 (1.53)
Peak of mean concentration (pg ml^−1^)				
Bolus Continuous	326 532	702 707	714 456	40 136
Peak of subject concentration (pg ml^−1^)				
Bolus Continuous	60–1297 212–1293	351–1856 303–1335	522–1124 170–683	20–105 57–376
Peak timing of mean concentration (pg ml^−1^)				
Bolus Continuous	1.5 3	2 4	2 4	3 4
Peak timing of subject concentration (pg ml^−1^)				
Bolus Continuous	1.5–2 3–4	2–3 4	1.5–3 3–4	2–3 3–6

*Note*: Abnormal concentrations (outliers) were not used to compute the mean data or standard deviation (SD). Individual subject concentrations were calculated from a sample size *n* = 20 for the bolus data from Janum et al. (2016), and *m* = 9 for the continuous infusion data from Berg et al. (2012). All concentrations were rounded to the nearest whole number except for the baseline measurements.

### 2.3 Data calibration

**FIGURE 3:**
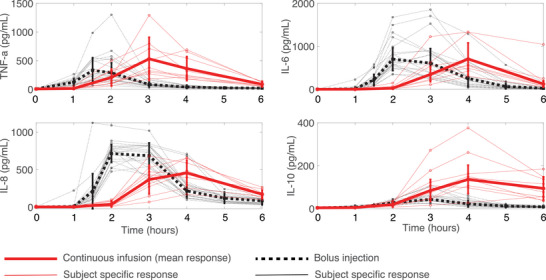
Mean and subject‐specific continuous infusion and bolus data. Red circles denote the continuous infusion data (m=9 subjects) connected by continuous lines, and black squares denote the bolus data (n=20 subjects) connected by dotted lines. Thin lines represent subject‐specific responses and thick vertical lines denote mean (SD).

To compare the cytokine responses from the two studies, we adjusted the bolus data so that both studies had the same baseline concentration. This was done by determining the difference ($\bar{d}$) between the mean bolus ($\bar{b}$) and continuous infusion ($\bar{c}$) baseline

$$\;{\bar{d}}_{0}^{j}={\bar{b}}_{0}^{j}\;-{\bar{c}}_{0}^{j}$$

for cytokine j= {TNF, IL6, IL8, IL10} and then shifting the concentrations by

$${\hat{b}}_{i}^{j}\;\left(k\right)=b_{i}^{j}\;\left(k\right)-{\bar{d}}_{0}^{j},$$

where $b_{i}^{j}\left(k\right)$ is the original cytokine concentration j at time i for the kth bolus participant (1≤k≤20). The adjusted cytokine concentration is denoted by ${\hat{b}}_{i}^{j}\left(k\right)$. Figure 3 displays the mean and subject‐specific continuous infusion and bolus data.

### 2.4 Mathematical model

**FIGURE 4:**
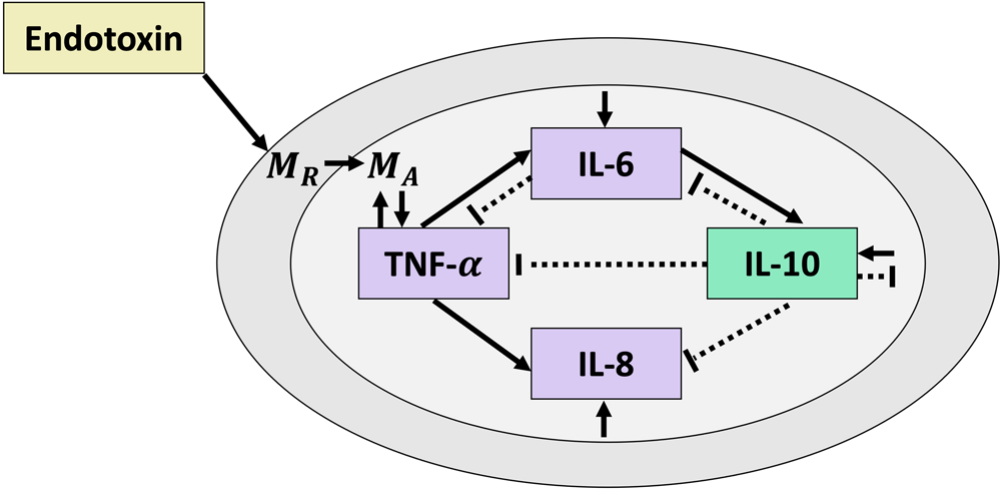
Endotoxin administration recruits (activates) monocytes from a large pool of resting monocytes ($M_{R}$). The active monocytes ($M_{A}$) upregulate the production of pro‐ (TNF‐α, IL‐6 and IL‐8) and anti‐ (IL‐10) inflammatory cytokines. TNF‐α generates positive feedback on monocyte, IL‐6 and IL‐8 production. IL‐6 exhibits anti‐inflammatory properties through self‐regulation, downregulation of TNF‐α, and upregulation of IL‐10. IL‐10 downregulates all cytokine production and the activation of monocytes. Solid black lines represent stimulation, and dotted black lines represent inhibition.

Our mathematical model (Figure 4)  adapted from our previous studies (Brady et al., 2018; Dobreva et al., 2021; Windoloski et al., 2023) predicting dynamics of the innate immune response to endotoxin included a system of seven ordinary differential equations (ODEs) with 45 parameters. The equations characterized the time‐varying endotoxin dose, the number of resting and activated monocytes, and concentrations of pro‐ and anti‐inflammatory cytokines. Below, we briefly describe the model and refer to Brady (2017) for a detailed derivation of the equations.

#### 2.4.1 Endotoxin

The equation determining the endotoxin concentration (E, ng kg^−1^) was adapted from Brady et al. (2018) and Dobreva et al. (2021) to account for a continuous infusion. This formulation is similar to Windoloski et al. (2023) and motivated by Day et al. (2006). The endotoxin rate of change was given by

(1) $$\frac{\mathrm{dE}}{\mathrm{dt}}=\left\{\begin{array}{c} D_{h}-k_{E}E,\;t\leq D_{\mathrm{ad}}\\ -k_{E}E,\;t\geq D_{\mathrm{ad}} \end{array}\right.,$$

where $D_{h}$ (ng kg^−1^ h^−1^) was the endotoxin dose administered per hour, $D_{\mathrm{ad}}$ (h) the dosing administration duration and $k_{E}$ (h^−1^) the endotoxin decay rate. The 2 ng kg^−1^ continuous infusion was administered over 4 h so $D_{h}=0.5,\;\;D_{\mathrm{ad}}=4,$ and E(0)=0, whereas $D_{h}=0,\;\;D_{\mathrm{ad}}=0,$ and E(0)=2 for the bolus injection.

#### 2.4.2 Monocyte and cytokines

The monocyte and cytokine interaction dynamics shown in Figure 4 were impacted through multiple feedback channels. The general structure of equations include an activation or production term, a source term, and a natural decay term. To capture the activation, we utilized Hill functions that describe the upregulation ($H_{Y}^{U}\left(X\right))$ or downregulation ($H_{Y}^{D}\left(X\right))$ of state Y by state X. This was modeled as

$$H_{Y}^{U}\;\left(X\right)=\frac{X^{h}}{\eta_{YX}^{h}+X^{h}},\;H_{Y}^{D}\;\left(X\right)=\frac{\eta_{YX}^{h}}{\eta_{YX}^{h}+X^{h}},$$

where h represents the steepness of the curve and $\eta_{YX}$ the half‐maximum value.

#### 2.4.3 Monocytes

During the endotoxin challenge, resting monocytes circulating in the blood are activated, upregulating cytokine production. This process regulates inflammation via positive and negative feedback (Rossol et al., 2011). The resting ($M_{R}$) and activated ($M_{A}$) monocytes (number of cells, noc) were found from

(2) $$\begin{array}{ccc} \frac{dM_{R}}{\mathrm{dt}} & = & k_{\mathrm{MR}}\;M_{R}\left(1-\frac{M_{R}}{M_{\infty }}\right)\\ & & -\;H_{M}^{U}\left(E\right)\left(k_{M}+k_{\mathrm{MTNF}}H_{M}^{U}\left(\mathrm{TNF}\right)\right)H_{M}^{D}\left(\mathrm{IL}10\right)M_{R} \end{array}$$

(3) $$\frac{dM_{A}}{\mathrm{dt}}=H_{M}^{U}\;\left(E\right)\left(k_{M}+k_{\mathrm{MTNF}}H_{M}^{U}\left(\mathrm{TNF}\right)\right)H_{M}^{D}\left(\mathrm{IL}10\right)M_{R}-k_{\mathrm{MA}}M_{A},$$

where $k_{\mathrm{MR}}$ (h^−1^) denoted the regeneration rate and $M_{\infty }$ (noc) the carrying capacity for the resting monocytes. The activated monocytes were upregulated by endotoxin (Rossol et al., 2011) at rate $k_{M}$ (h^−1^) and the inflammatory cytokine TNF‐α at rate $k_{\mathrm{MTNF}}\;$(h^−1^). They were also downregulated by anti‐inflammatory cytokine IL‐10 (Kucharzik et al., 1998). This process was activated relative to the resting monocytes. The increase in activated monocytes caused an identical decrease in the resting monocytes. Finally, the activated monocytes decayed at rate $k_{\mathrm{MA}}$ (h^−1^).

#### 2.4.4 Inflammatory mediators

Activated monocytes upregulate cytokines, which are signalling proteins that promote or suppress inflammation (Murphy, 2012). Cytokines that stimulate inflammation, called pro‐inflammatory, include TNF‐α, IL‐6 and IL‐8 (Johnston & Webster, 2009). TNF‐α is an early pro‐inflammatory mediator responsible for the induction of fever (Murphy, 2012) and the recruitment of other pro‐inflammatory cytokines (Johnston & Webster, 2009). IL‐6 is a secondary pro‐inflammatory mediator primarily involved in the induction of the liver acute phase response (Johnston & Webster, 2009). However, it can also exhibit anti‐inflammatory properties (Tilg et al., 1994). IL‐8 is a late pro‐inflammatory mediator mainly responsible for recruiting neutrophils to the target site (Bickel, 1993). Monocytes also release anti‐inflammatory cytokines, particularly IL‐10, to counteract pro‐inflammatory responses and provide a balanced immune response (Johnston & Webster, 2009). These four cytokines are essential components of the innate immune response. Their interactions can be predicted by

(4) $$\frac{d\mathrm{TNF}}{\mathrm{dt}}=k_{\mathrm{TNFM}}\;H_{\mathrm{TNF}}^{D}\left(\mathrm{IL}6\right)H_{\mathrm{TNF}}^{D}\left(\mathrm{IL}10\right)M_{A}-k_{\mathrm{TNF}}\left(\mathrm{TNF}-w_{\mathrm{TNF}}\right)$$

(5) $$\frac{\mathrm{dIL}6}{\mathrm{dt}}=\left(k_{6M}+k_{6\mathrm{TNF}}H_{\mathrm{IL}6}^{U}\left(\mathrm{TNF}\right)\right)H_{\mathrm{IL}6}^{D}\left(\mathrm{IL}6\right)H_{\mathrm{IL}6}^{D}\left(\mathrm{IL}10\right)M_{A}-k_{6}\left(\mathrm{IL}6-w_{6}\right)$$

(6) $$\frac{d\mathrm{IL}8}{\mathrm{dt}}=\left(k_{8M}+k_{8\mathrm{TNF}}H_{\mathrm{IL}8}^{U}\left(\mathrm{TNF}\right)\right)H_{\mathrm{IL}8}^{D}\left(\mathrm{IL}10\right)M_{A}-k_{8}\left(\mathrm{IL}8-w_{8}\right)$$

(7) $$\frac{d\mathrm{IL}10}{\mathrm{dt}}=\left(k_{10M}+k_{106}H_{\mathrm{IL}10}^{U}\left(\mathrm{IL}6\right)\right)M_{A}-k_{10}\left(\mathrm{IL}10-w_{10}\right).$$

In Equation (4), TNF‐α was activated by monocytes (Johnston & Webster, 2009) at rate $k_{\mathrm{TNFM}}\;$(pg ml^−1^ h^−1^ noc^−1^) and downregulated by IL‐6 and IL‐10 (Tilg et al., 1994). In Equation (5), IL‐6 was activated by monocytes and TNF‐α (Johnston & Webster, 2009) at rates $\;k_{6M}$ (pg ml^−1^ h^−1^ noc^−1^) and $k_{6\mathrm{TNF}}$ (pg ml^−1^ h^−1^ noc^−1^), and downregulated by itself (Verboogen et al., 2019) and IL‐10 (Johnston & Webster, 2009). Similarly in Equation (6), IL‐8 was activated by monocytes and TNF‐α at rates $k_{8M}$ (pg ml^−1^ h^−1^ noc^−1^) and $k_{8\mathrm{TNF}}$ (pg ml^−1^ h^−1^ noc^−1^), and downregulated by IL‐10 (Johnston & Webster, 2009). In Equation (7), IL‐10 was activated by monocytes and IL‐6 at rates $k_{10M}$ (pg ml^−1^ h^−1^ noc^−1^) and $k_{106}$ (pg ml^−1^ h^−1^ noc^−1^), (Jin et al., 2013; Murphy, 2012). Cytokines decayed to their baseline concentrations $w_{i}$ (pg ml^−1^) at rate $k_{i}$ (h^−1^) for i={TNF,6,8,10}.

#### 2.4.5 Model summary

The mathematical model consisted of an ODE system of the form

(8) $$\frac{\mathrm{dX}}{\mathrm{dt}}=\;f\left(t,X,\theta \right),$$

where $X\in R^{7}$ denoted the time‐varying states X={E,MR,MA,TNF,IL6,IL8,IL10} determining endotoxin (E), monocytes (resting $M_{R}$ and activated $M_{A}$), TNF‐α, IL‐6, IL‐8 and IL‐10 concentrations. The model parameters $\theta \in R^{45}$ are listed in the Appendix Table A1 with subsections indicating what state the parameters belong to.

To fit the mathematical model to the experimental data, we minimized the least squares cost function $J=r^{T}r,$ where $r=[r_{\mathrm{TNF}}\;r_{\mathrm{IL}6}\;r_{\mathrm{IL}8}\;r_{\mathrm{IL}10}]\;$ is the residual vector and

(9) $$r_{k}=\frac{1}{\sqrt{N}}\;\left(\frac{\left[y_{1}^{k}\text{…}y_{N}^{k}\right]-y_{data}^{k}}{\max(y_{data}^{k})}\right),\;k=\left\{\mathrm{TNF},\mathrm{IL}6,\mathrm{IL}8,\mathrm{IL}10\right\}.$$

Here N refers to the number of data points for each state k, $y_{i}^{k}=g\left(t_{i},X_{k}\left(t_{i}\right);\theta \right)$ denotes the model output for the state k at time $t_{i}$ for 1≤i≤N, and $y_{\mathrm{data}}^{k}$ is the associated data. The least squares cost J was minimized using *fmincon* from MATLAB (The MathWorks Inc., Natick, MA, USA). Upper and lower parameter bounds were set by multiplying and dividing the parameters’ nominal value by a factor of 4.

### 2.5 Nominal parameters

Nominal parameter values were taken from Brady (2017) except for cytokine baseline concentrations ($w_{\mathrm{TNF}},w_{6},w_{8}\;$ and $w_{10}$), which were set to the mean continuous infusion and bolus data. We manually adjusted nominal parameters impacting peak timing to account for the observation (Figure 2) that the timing of cytokine activation depends on the administration method. To support the convergence of the gradient‐based optimizer used to estimate identifiable model parameters, we further improved the nominal model fit to the peak magnitudes of cytokine profiles by scaling the peak cytokine concentrations as described in detail in Windoloski et al. (2023) and Windoloski (2023). Scaling analysis was applied to all simulations to capture mean and individual variation in cytokine responses. A scaling threshold was included for subject‐specific dynamics to reduce the number of scaled parameters for individual subjects. Appendix Table A1 lists the nominal parameters for the continuous infusion and bolus mean model.

Nominal parameter values used for model calibration to the individual data were set to the mean optimal values except for the initial cytokine concentrations ($w_{\mathrm{TNF}},w_{6},w_{8},\;w_{10}$), which were set to the individual's cytokine value at baseline. For subjects missing measurements at time zero, we scaled their concentration after 1 h based on values from subjects where data were available; IL‐6 and IL‐10 baseline values were set at 50% and 75% of their concentrations at hour 1.

### 2.6 Sensitivity analysis and subset selection

The highly nonlinear mathematical model had seven states and 45 parameters. Because of its structure (Brady, 2017; Brady et al., 2018) and the quantity of data, we selected a parameter subset from the rate constants to estimate. We first conducted a local relative sensitivity analysis as described in Olufsen and Ottesen (2013) and Pope et al. (2009) on the mean continuous infusion response using the residual vector in Equation (9). The sensitivity matrix χ was given by

(10) $$\chi =\frac{\partial r}{\partial \log\left(\theta \right)}=\frac{\partial y}{\partial \theta }\;\frac{\theta }{\max\left(y_{\mathrm{data}}\right)},$$

where y=g(t,X(t),θ) was the model output at time t, θ the nominal parameter set, and $y_{\mathrm{data}}$ the mean continuous infusion data. We ranked relative sensitivities by computing the two norm of each column of χ, obtaining a single sensitivity per parameter. We repeated the sensitivity analysis by simulating 100 runs sampling parameters from a uniform distribution varying ±30% around the parameter's nominal value to study effects due to perturbations in parameter values.

Sensitive rate constants were used to select an identifiable parameter subset that can be estimated. We utilized two practical identifiability techniques, the structured correlation method (SCM) and the singular value decomposition followed by QR factorization (SVD‐QR) method (Miao et al., 2011; Olufsen & Ottesen, 2013) (described in detail in the Supporting Information). The subset selection methods determine identifiable parameters near the nominal values. To ensure that optimal values were also identifiable, we conducted 20 optimizations for each subset with nominal parameters drawn from a uniform distribution of ±10% of each estimated parameter's nominal value. All other parameters were fixed. For each of the 20 runs, we calculated the coefficient of variation ($\mathrm{CoV}=\sigma_{i}\;/{\bar{\theta }}_{i}$), where the mean and standard deviation for the estimated parameters were denoted by $\bar{\theta_{i}}$ and $\sigma_{i}$. Parameters in each subset with CoV≥0.1 were identified. The least sensitive parameter was removed from the set, and this process was repeated until all estimated parameters in each subset had a CoV<0.1. The resulting parameter subsets were considered sensitive and identifiable and used for parameter estimation.

### 2.7 Statistical methods

For each parameter subset, the goodness of fit was computed using the coefficient of determination ($R^{2}$) (Dodge, 2008), the corrected Akaike information criterion (AICc) (Burnham & Anderson, 2002) and the Bayesian information criterion (BIC) (Schwarz, 1978). Details of these measurements are provided in the Supporting Information.

We also computed parameter and model confidence and prediction intervals using the frequentist approach detailed in Banks et al. (2009), Seber and Wild (2003), and Smith (2014) and the Supporting Information. Parameter confidence intervals for optimized parameter $\;{\tilde{\theta }}_{i}$ were computed as

(11) $${\tilde{\theta }}_{i}\pm t_{N-q}^{\alpha /2}\sqrt{\Sigma_{ii}},$$

where N was the total number of data points, q was the number of parameters that were estimated, $t_{N-q}^{\alpha /2}$ was the *t*‐value from Student's *t*‐distribution for confidence level 1−α with N−q degrees of freedom, and Σ was the variance estimator matrix. The asymptotic prediction interval for cytokine k at time $t_{i}$ was given by

(12) $${\mathrm{PI}}_{i}^{k}={\tilde{y}}_{i}^{k}\;\pm t_{N_{k}-q_{k}}^{\alpha /2}s_{k}\sqrt{1+G_{\mathrm{ik}}^{T}{\left(\chi_{k}^{T}\left(\tilde{\theta }\right)\chi_{k}\left(\tilde{\theta }\right)\right)}^{-1}G_{\mathrm{ik}}}$$

and the confidence interval by

(13) $${\mathrm{CI}}_{i}^{k}={\tilde{y}}_{i}^{k}\;\pm t_{N_{k}-q_{k}}^{\alpha /2}s_{k}\sqrt{G_{\mathrm{ik}}^{T}{\left(\chi_{k}^{T}\left(\tilde{\theta }\right)\chi_{k}\left(\tilde{\theta }\right)\right)}^{-1}G_{\mathrm{ik}}}.$$

Here ${\tilde{y}}_{i}^{k}$ was the model output computed with optimal parameter vector $\tilde{\theta }$, $N_{k}$ was the number of data points for cytokine k, $q_{k}$ was the number of estimated parameters that impacted cytokine state k, and $t_{N_{k}-q_{k}}^{\alpha /2}$ was the *t*‐value for confidence level 1−α with $N_{k}-q_{k}\;$ degrees of freedom. The matrix $\chi_{k}\left(\tilde{\theta }\right)$ represents the optimal model's sensitivity matrix for cytokine k, which is like Equation (10) and formally defined in the Supporting Information. Note that columns in $\chi_{\mathrm{TNF}}\left(\tilde{\theta }\right),\;\chi_{\mathrm{IL}6}\left(\tilde{\theta }\right)$ and $\chi_{\mathrm{IL}10}\left(\tilde{\theta }\right)$ corresponding to IL‐8 parameters were eliminated since they did not impact those state variables (see Figure 4). Entries of these columns were approximately zero and made $F_{k}=\chi_{k}^{T}\;\left(\tilde{\theta }\right)\chi_{k}\left(\tilde{\theta }\right)$ singular unless removed. The matrix $G_{ik}^{T}\;$ was defined as

(14) $$G_{ik}^{T}=\left(\frac{\partial {\tilde{y}}_{i}^{k}}{\partial \theta_{1}},\frac{\partial {\tilde{y}}_{i}^{k}}{\partial \theta_{2}},\text{…},\;\frac{\partial {\tilde{y}}_{i}^{k}}{\partial \theta_{q_{k}}}\;\right),$$

which was ith row of the submatrix $\chi_{k}\left(\tilde{\theta }\right)$, and the variance estimator $s_{k}^{2}$ was given by

(15) $$s_{k}^{2}=\frac{1}{N_{k}-q_{k}}\;r_{k}^{T}r_{k},$$

with $r_{k}$ defined as the difference between the model and data for cytokine k. Due to the small number of data points per cytokine, we generated pseudo data to compute uncertainty intervals. Data points at 8 and 12 h were set by quartering the cytokine concentration at 6 h and returning the cytokine to the baseline value. Then, a piecewise cubic spline interpolation was performed from t=0 to t=12 h.

Statistical data analysis included a two‐sample unequal variances *t*‐test (α=0.05) on the continuous infusion and bolus data before data calibration to compare their maximal concentrations and peak timing statistically. Abnormal cytokine responses (outliers in Figure 2) were omitted from the data sampled to conduct the hypothesis test, and the data distributions were approximately normal. A two‐sample unequal variances *t*‐test (α=0.05) was also performed on the set of optimized parameters from subject‐specific optimizations to determine statistically significant differences in parameter values between the two administration methods. Parameter values that were outliers within their data set were not included in the data sampled to conduct the hypothesis test. The distributions of the optimal parameter values were approximately normal.

## 3 RESULTS

### 3.1 Data

Statistical comparison (Table 2) of the continuous infusion and bolus injection data shows a significantly smaller peak concentration of IL‐8 (P=0.00147) and a larger peak concentration of IL‐10 (P=0.00200) with continuous infusion. The peak concentration for TNF‐α (P=0.0809) and IL‐6 (P=0.702) did not differ significantly between the two studies, but the time to peak cytokine concentration was considerably longer for all cytokines during the continuous infusion study: TNF‐α, IL‐6 and IL‐8 (P<0.0001) and IL‐10 (P=0.00695).

**TABLE 2:** Statistical significance (α=0.05) of data attributes between the continuous infusion (*m* subjects) and bolus (*n* subjects) studies in Berg et al. (2012) and Janum et al. (2016).

Cytokine	Maximal concentration	Time to maximal concentration
TNF‐α	P=0.0809(m=9,n=19)	P<0.0001(m=9,n=19)
IL‐6	P=0.702(m=9,n=18)	P<0.0001(m=9,n=18)
IL‐8	P=0.00147(m=9,n=19)	P<0.0001(m=9,n=19)
IL‐10	P=0.00200(m=8,n=17)	P=0.00695(m=8,n=18)

Subjects with abnormal responses (outliers) were omitted from the sample.

### 3.2 Sensitivity analysis and subset selection

**FIGURE 5:**
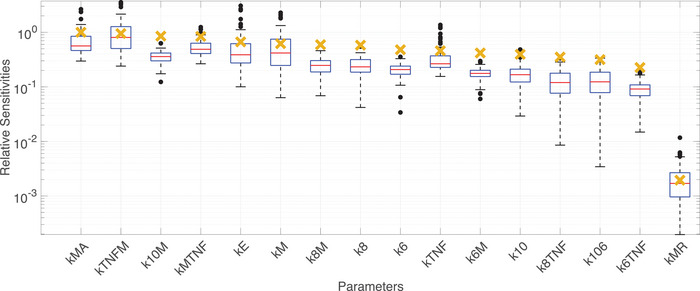
Ranked sensitivities. Local sensitivities, scaled by the maximum sensitivity, are denoted by yellow crosses. Boxplots of scaled relative sensitivities are generated from n=100 local sensitivity analysis simulations. Values are scaled by the maximum average sensitivity. Black circles denote outliers.

Single and repeated sensitivity analysis (Figure 5) highlighted the system's dependence on endotoxin, activated monocytes, TNF‐α and IL‐10 dynamics. The system's most sensitive parameters were the growth or decay of these states, where$\;k_{\mathrm{TNFM}}$ (the growth rate of TNF‐α by monocytes) and $k_{\mathrm{MA}}$ (the activated monocyte decay rate) have the most significant impact on the model. This can be explained by endotoxin and activated monocytes promoting the activation of cytokines, where TNF‐α and IL‐10 are the main cytokines that upregulate and downregulate other states. The least sensitive rate constant is $k_{\mathrm{MR}}$, the regeneration rate for resting monocytes. Given that our study administered a finite dose of endotoxin that did not deplete the resting monocytes before the system can recover, it is reasonable that this parameter has a minute effect on the system. The single and repeated sensitivity analysis results exhibited similar behaviour with minor differences in the ranked order of sensitivity. This observation and careful scaling of nominal parameter values provide a good foundation for choosing identifiable subsets among the sensitive parameters.

The parameter $k_{\mathrm{MR}}$ was insensitive, and was removed from the subset and fixed at its nominal value. Identifiability analysis using the SCM and SVD‐QR method was performed on the remaining 15 sensitive rate constants. Because different identifiability analysis methods are not guaranteed to produce the same results (Brady, 2017), we generated three parameter subsets, two using only the SCM and one using the SVD‐QR followed by the SCM. Results showed that all rate constants for monocytes, IL‐6, IL‐8 and IL‐10 cannot be uniquely estimated. Therefore, the sensitive rate constants were split into two subsets, one that included monocyte‐activated growth rates and another that contained cytokine‐activated growth rates. The SCM resulted in the two subsets:

$$S_{1}=\left\{k_{E},k_{\mathrm{MA}},k_{\mathrm{MTNF}},k_{\mathrm{TNF}},k_{\mathrm{TNFM}},k_{6},k_{6\mathrm{TNF}},k_{8},k_{8\mathrm{TNF}}\right\}$$

$$S_{2}=\left\{k_{\mathrm{MA}},k_{M},k_{\mathrm{TNF}},k_{\mathrm{TNFM}},k_{6},k_{6M},k_{8},k_{10},k_{10M}\right\}.$$

The third subset was found by performing SVD‐QR followed by the SCM on all 15 rate constants, which resulted in the subset:

$$S_{3}=\left\{k_{\mathrm{MA}},k_{\mathrm{TNF}},k_{\mathrm{TNFM}},k_{6},k_{6M},k_{8},k_{8M},k_{10M}\right\}.$$

The identifiability and convergence of the above parameter subsets were checked numerically using the coefficient of variation method, enabling us to reduce the subsets further. We obtained three sensitive and identifiable parameter subsets:

$$s_{1}=\left\{k_{\mathrm{MA}},k_{\mathrm{MTNF}},k_{\mathrm{TNF}},k_{\mathrm{TNFM}},k_{6},k_{8},k_{8\mathrm{TNF}}\right\}$$

$$s_{2}=\left\{k_{\mathrm{MA}},k_{M},k_{\mathrm{TNF}},k_{\mathrm{TNFM}},k_{6},k_{6M},k_{8},k_{10},k_{10M}\right\}$$

$$s_{3}=\left\{k_{\mathrm{MA}},k_{\mathrm{TNF}},k_{\mathrm{TNFM}},k_{8},k_{8M},k_{10M}\right\}.$$

Note that $S_{2}=s_{2}$, indicating that the subset $S_{2}$ was identifiable.

### 3.3 Parameter estimation and uncertainty quantification

Model fit for the mean continuous infusion data ($R^{2}$, AICc, BIC and least squares cost J) for subsets $s_{1},s_{2}$ and $s_{3}$ are reported in Table 3. Subset $s_{3}$ had the lowest AICc and BIC values, but the $R^{2}$ value and least squares cost did not differ significantly between the three subsets. Given the significance of the AICc and BIC values, we conduct the remaining simulations using $S_{\mathrm{Final}}=s_{3}=\left\{k_{\mathrm{MA}},k_{\mathrm{TNF}},k_{\mathrm{TNFM}},k_{8},k_{8M},k_{10M}\right\}$.

**TABLE 3:** Goodness of fit measurements for the optimized subsets $s_{1},s_{2}$ and $s_{3}$.

Subset Estimated	Number of Parameters	Average $R^{2}$	AICc	BIC	J
*s*_1_	7	0.915	15.4	16.6	0.0602
*s*_2_	9	0.923	24.7	22.5	0.0466
*s*_3_	6	0.913	11.1	13.3	0.0547

*Note*: The coefficient of determination is denoted as *R* ^2^. AICc, corrected Akaike information criterion; BIC Bayesian information criterion; *J*, least squares cost.

**FIGURE 6:**
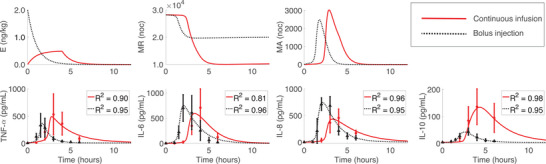
Optimized model fit to mean continuous infusion and bolus data estimating $S_{\mathrm{Final}}$. The mean continuous infusion fit is marked by red continuous lines, and the mean (SD) of the data by red circles and error bars. The mean bolus fit has black dotted lines, and black triangles and error bars denote the mean (SD) of the data.

The mean continuous infusion model exhibited later activation of monocytes and cytokines compared to the bolus injection model (Figure 6). As a result, the main pro‐ and anti‐inflammatory cytokines TNF‐α and IL‐10 had larger peak concentrations. The immune resolution time during the continuous infusion model was approximately 10–12 h, whereas the mean bolus model was only 6–8 h. Comparison of model fits by the coefficient of determination ($R^{2}$) for each cytokine revealed that TNF‐α and IL‐6 were fitted better by the bolus model, while the continuous infusion model better predicted IL‐8 and IL‐10. Differences were minor, though, specifically for IL‐8 and IL‐10. The unobserved model states $E,M_{R}$ and $M_{A}$ align with dynamics suggested or observed in both experimental and modelling studies, indicating that we captured appropriate time course dynamics for these states. We used an endotoxin decay rate almost identical to that of Chow et al. (2005) and the resting monocyte depletion followed a similar time course to that reported by Kiers et al. (2017). Furthermore, the resting monocyte recovery time course for our model (shown in Figure S36 in the Supporting Information) is similar to the resting period between trial days in the study by Janum et al. (2016), suggesting that it could take at least 4 weeks for complete immune recovery.

**FIGURE 7:**
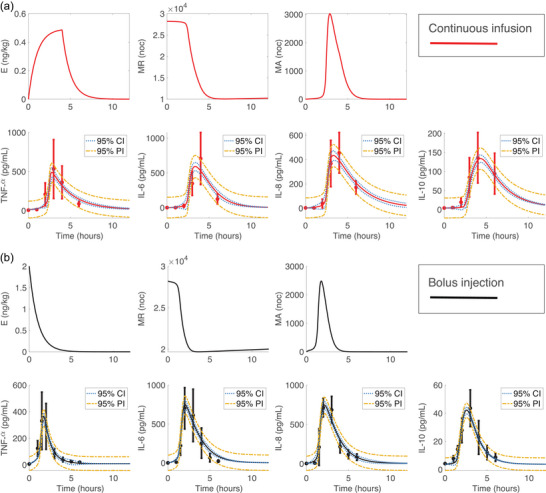
Ninety‐five percent prediction and confidence intervals for the mean (a) continuous infusion and (b) bolus responses. Mean and SD data points are marked by circles and error bars. Prediction intervals are the yellow dashed–dotted lines, and confidence intervals are the blue dotted lines.

We generated N=61 data points for each cytokine to determine the mean data and model uncertainty using confidence level (1−α) with α=0.05. Confidence bounds on the optimal parameters from the continuous infusion and bolus mean model responses are given in Table 4. The upper and lower bounds remained within the physiological values (positive parameters close to their nominal value) except for $k_{10M}$, which had a negative lower bound. Prediction and confidence intervals on the optimal mean model are shown in Figure 7. Both prediction and confidence intervals for the bolus (Figure 7b) were tighter than those for the continuous infusion model (Figure 7a), indicating the variability of mean measurements and model output was larger in the continuous infusion data. This is plausible, given the sample sizes of the two studies. The lower bound for the prediction intervals of both dose types extended into negative cytokine values, which is mathematically but not physiologically appropriate. The negative portion of the lower bound could be omitted to provide a positive lower bound of the prediction interval. If additional data were available or computationally generated for these cytokines, then the prediction bounds would be tighter and, eventually, non‐negative. Similar uncertainty results (seen in Figures S37–S43 in the Supporting Information) were obtained using Bayesian inference with the delayed rejection adaptive metropolis (DRAM).

**TABLE 4:** Optimal 95% parameter confidence bounds for the mean continuous infusion and bolus model.

Parameter	Continuous infusion (optimal value ± bound)	Bolus (optimal value ± bound)
*k*_MA_	3.49 ± 0.0994	2.67 ± 0.0787
*k*_TNF_	0.423 ± 0.132	1.40 ± 0.118
*k*_TNFM_	1.39 ± 0.0696	0.998 ± 0.0398
*k*_8_	0.386 ± 0.119	0.686 ± 0.100
*k*_8M_	0.613 ± 0.193	0.746 ± 0.163
*k*_10M_	0.0365 ± 0.127	0.0150 ± 0.124

**FIGURE 8:**
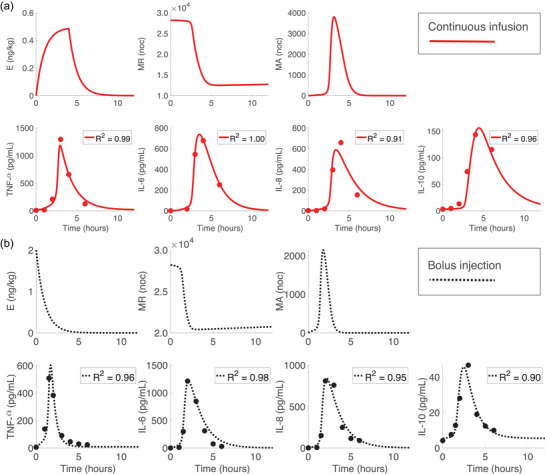
Optimal model responses for (a) subject 1 from the continuous infusion study and (b) subject 16 from the bolus study.

We fitted the model to the subject‐specific cytokine profiles from the continuous infusion (m=9) and bolus injection (n=20) studies by estimating the parameters in $S_{\mathrm{Final}}$. Results for continuous infusion subject 1 and bolus injection subject 16 are shown in Figure 8, and dynamics for the remaining subjects are presented in Figures S1–S29 in the Supporting Information. Results showed that our model captures varying cytokine responses to the same total dose of endotoxin for both administration methods. While individual peak cytokine concentrations and peak timing differed from that in the mean response, the model (shown in Figure 8a, b) is sufficiently robust to capture variation in data. This is evidenced by high $R^{2}$ values for all subjects.

**FIGURE 9:**
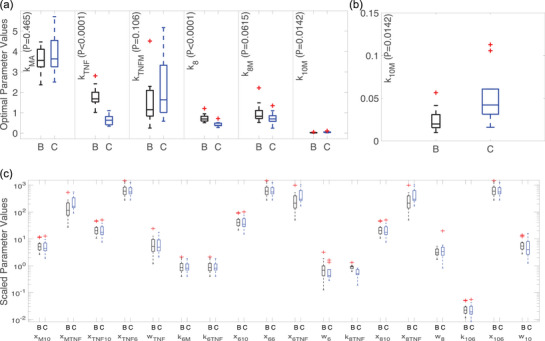
(a) Boxplots of subject‐specific optimized parameters from the bolus ‘B’ (black, n=20 subjects) and continuous ‘C’ (blue, m=9 subjects) administration models. Associated *P*‐values are listed next to each parameter. (b) Zoomed‐in boxplot of optimized parameter $k_{10M}$ from (a). (c) Boxplots of subject‐specific scaled parameters. On all plots outliers are denoted by the red cross. Parameters considered statistically significant (α=0.05) include $k_{\mathrm{TNF}}\;$(P<0.0001), $k_{8}$ (P<0.0001), and $k_{10M}$ (P=0.0142). Parameters not statistically significant include $k_{\mathrm{MA}}$ (P=0.465), $k_{\mathrm{TNFM}}$ (P=0.106), and $k_{8M}$ (P=0.0615). This figure was generated using MATLAB code adapted from Danz (2023).

The mean and standard deviation for subject‐specific optimal and scaled parameters are listed in the Appendix Table A2, and a boxplot of the optimized subject‐specific parameter values is shown in Figures 9a, b. We observed similar median parameter values for the continuous infusion and bolus subject‐specific optimizations for parameters $k_{\mathrm{MA}},k_{\mathrm{TNFM}}$ and $k_{8M}$. For parameters $k_{\mathrm{MA}},k_{\mathrm{TNFM}}$ and $k_{10M}$, there was larger variation in the continuous infusion than the bolus injection. Optimized parameter values denoted as outliers in Figure 9a, b correspond to subjects 3, 5 and 9 from the continuous infusion study and subjects 3, 9, 13, 14 and 20 from the bolus study. These subjects all had abnormal endotoxin responses (at least one outlying data point in Figure 2). Boxplots of all scaled subject‐specific parameters are shown in Figure 9c.

Statistical comparison of the continuous infusion and bolus optimized parameters showed that $k_{\mathrm{TNF}}$ and $\;k_{8}\;$ (P<0.0001) were significantly larger during the bolus injection, indicating the TNF‐α and IL‐8 decayed faster during the bolus dose. Additionally, $k_{10M}$ (P=0.0142) was significantly larger during the continuous infusion, implying that monocyte activation of IL‐10 was more pronounced during a continuous infusion of endotoxin. As a result, the continuous infusion had a significantly larger activation response of IL‐10 by monocytes and substantially smaller TNF‐α and IL‐8 degradation rates. Parameters $k_{\mathrm{MA}}$ (P=0.465), $k_{\mathrm{TNFM}}$ (P=0.106) and $\;k_{8M}$ (P=0.0615) were not significantly different between the two administration methods. Abnormal responses denoted as outliers in Figure 9 were not included in the sample from each study.

### 3.4 Infusion perturbations

**FIGURE 10:**
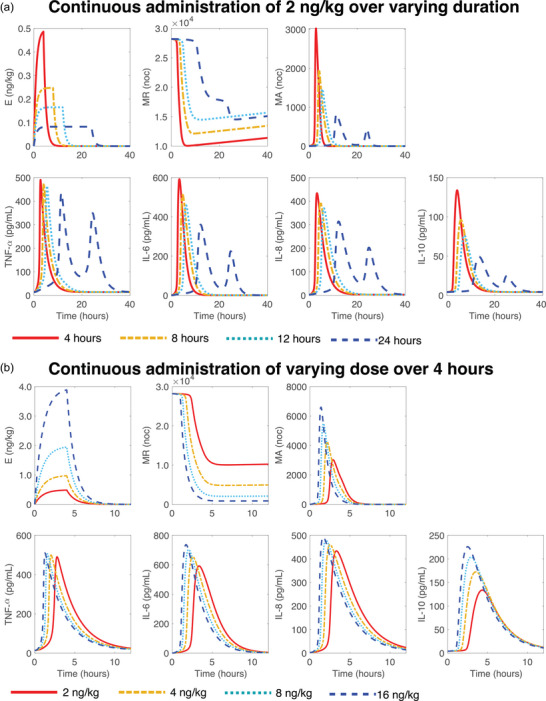
Continuous infusion mean model response when (a) 2 ng kg^−1^ of endotoxin is administered as a 4 (red solid lines), 8 (yellow dashed‐dotted lines), 12 (light blue dotted lines), and 24‐h (dark blue dashed lines) continuous infusion, and (b) 2 (red solid lines), 4 (yellow dashed‐dotted lines), 8 (light blue dotted lines), and 16 ng kg^−1^ (dark blue dashed lines) of endotoxin is administered as a 4‐h continuous infusion.

We used the optimal mean continuous infusion model to study the response to a longer duration of inflammation and enhanced immune stimulation. Figure 10a shows the model response when 2 ng kg^−1^ of endotoxin was given continuously over 4, 8, 12 and 24 h. The infusion duration impacts peak cytokine concentrations and the response's resolution time. Peak concentrations declined and occurred later as the infusion duration increased. Cytokine concentrations returned to baseline approximately 12, 16, 20 and 36 h following the infusion start for the 4, 8, 12 and 24 h continuous infusions. The system exhibited oscillatory behaviour when the infusion was extended to 24 h. The increase of anti‐inflammatory cytokine IL‐10 around 10 h combated the initial pro‐inflammatory response of TNF‐α to decline around 12 h. However, because the endotoxin was still being administered, it rebounded a second time once IL‐10 levels began to decline. Following the termination of endotoxin administration, the monocytes were no longer activated, and as a result, the inflammatory markers returned to baseline. This recurrent inflammatory behaviour transpired when endotoxin was administered for 20 to 32 h, after which the stimulation from the endotoxin was not strong enough to induce a pronounced response (Figure S35 in the Supporting Information). This simulation also shows that the system takes approximately 21–23 days to recover (Figure S36 in the Supporting Information) relative to the resting monocyte population returning to the baseline value.

Figure 10b displays the model response for a 4 h continuous endotoxin infusion of 2, 4, 8 and 16 ng kg^−1^. The total endotoxin dose impacted peak cytokine concentrations and the immune resolution time. Larger doses of endotoxin resulted in earlier, greater peak cytokine concentrations, which occurred approximately 1.5–2 h before peak cytokine concentrations for smaller endotoxin doses. Additional simulations increasing both the duration of the continuous infusion and the total dose of endotoxin are shown in Figures S30–S34 the Supporting Information.

## 4 DISCUSSION

This study used hypothesis‐generating mathematical modelling to compare bolus and continuous administration of LPS. The model was calibrated to data from Berg et al. (2012) and Janum et al. (2016). Data analysis revealed that IL‐10 has a significantly higher peak for the continuous dose, while the peak IL‐8 concentration was higher with the bolus injection. For the 2 ng kg^−1^ 4‐h continuous dose, peak cytokine concentrations occurred significantly later than in the bolus dose. The model predicts that this delayed behaviour continues when the dose is given over a longer time. Model parameter analysis provides insight into what processes may change with administration methods. Our results suggest that the continuous infusion of endotoxin increases the monocyte production rate of anti‐inflammatory cytokine IL‐10 and decreases the clearance rates of significant pro‐inflammatory markers TNF‐α and IL‐8. Our continuous infusion model is crucial as it can replicate characteristics of clinical inflammation associated with prolonged elevation of immune markers when endotoxin infusion is extended and pronounced cytokine responses when the endotoxin dosage is increased. Interestingly, administration over 20 and 32 h produces double cytokine peaks, indicating inflammation recurrence. Finally, we found that it takes over 20 days before the resting monocytes have reached the same level as before the stimulus.

Our findings agree with observations in Kiers et al. (2017) comparing a 2 ng kg^−1^ bolus response to a 1 ng kg^−1^ bolus followed by a 3 ng kg^−1^ continuous infusion. A bolus injection followed by a continuous infusion showed higher IL‐10 production and prolonged symptoms due to an extended elevation of cytokines. They also reported significantly higher production of TNF‐α, IL‐6 and IL‐8 with a bolus injection plus continuous infusion. Our study exhibited increased TNF‐α production, though results were not statistically significant, likely due to the small number of subjects and high variance between them. IL‐8 had significantly lower peaks during the continuous infusion (Figure 6). Our findings also agree with the partial conclusion of Taudorf et al. (2007), who reported that (i) the release of cytokines TNF‐α and IL‐6 occurred significantly later with the continuous infusion compared to a bolus injection and (ii) TNF‐α and IL‐6 concentrations were significantly higher for the bolus dose. For our study, IL‐6 was higher for the bolus dose, but again, the results were not significant. Taudorf et al. (2007) also reported higher neutrophil concentrations that peaked earlier with the bolus dose. Our study did not account for neutrophil dynamics, a component that could be added in future studies. Differences in our findings could result from low endotoxin dosage in Taudorf et al. (2007) and unequal total endotoxin dosing in Kiers et al. (2017). Overall, our findings implicate that continuous system stimulation over hours could promote a more significant anti‐inflammatory response to counteract prolonged levels of pro‐inflammatory cytokines, leading to lower maximal concentrations of secondary cytokines such as IL‐8.

A significant contribution of this study is the statistical analysis of optimal parameter distributions between the two administration methods, which has not been examined in earlier works. Results show that the activation rate of IL‐10 by monocytes was significantly larger, and the TNF‐α and IL‐8 decay rates were substantially lower in the continuous infusion versus the bolus injection. These results suggest that continual endotoxin infusion amplifies the monocyte production of IL‐10 and dulls the resolution of pro‐inflammatory cytokines TNF‐α and IL‐8. Because endotoxin is rapidly degraded in the liver (Mathison & Ulevitch, 1979), a bolus of endotoxin results in short‐lived exposure to resident macrophages in the liver while a continuous infusion elicits a longer, more steady exposure that could allow for adaptive mechanisms to set in, such as decreased cytokine clearance. Therefore, given that TNF‐α and IL‐8 are mainly cleared by receptor‐mediated endocytosis and renal elimination (Bemelmans et al., 1996; Matsushima et al., 2022; Rennen et al., 2003; Zeilhofer & Schorr, 2000), reduced receptor expression from the continuous endotoxin exposure could result in lower clearance rates of TNF‐α and IL‐8. While our results suggest physiological differences in processes relating to stimulation and degradation rates, where a continuous infusion may allow mechanisms to adapt that are otherwise not present during the short‐lived bolus administration, our model is a simplification of the immune system. There are physiological processes and mechanisms that are not explicitly modelled in our study, so the impact of those processes may be accounted for in our model parameters. Hence, additional studies are needed to verify our results.

Kiers et al. (2017) indicated that a continuous infusion of endotoxin is a more probable model of prolonged inflammation in conditions like sepsis, where a hyperinflammatory state (often referred to as a cytokine storm) is accompanied by an immunosuppressive phase with elevated anti‐inflammation levels (Nedeva et al., 2019; P. Torres et al., 2022). Thus, stimulation by a continuous infusion of endotoxin may exhibit mild but clear signs of a prolonged pro‐inflammatory response and a hyperactive anti‐inflammatory response, similar to dynamics observed in sepsis and supporting the hypothesis of Kiers et al. (2017). We note, however, that LPS studies cannot fully represent the dynamics of sepsis since LPS stimulates the toll‐like receptor 4 (TLR‐4) pathway (Zamyatina & Heine, 2020), whereas inflammation during sepsis can be stimulated via multiple pathways (Zhang & Ning, 2021).

The model analysis demonstrated the reliance of dynamics on the endotoxin, monocyte, TNF‐α and IL‐10 states. These constituents encompass primary elements of the inflammatory response—immune cells that respond to stimuli and the main pro‐ and anti‐inflammatory cytokines that modulate the response strength (Johnston & Webster, 2009). Thus, it is plausible that these components strongly dictate immune dynamics. It is also reasonable that the least influential component is the monocyte regeneration rate since the challenges analysed here are from short‐lived low‐dose endotoxin exposure, and the monocyte pool is not depleted before endotoxin clearance. However, we suspect that this parameter is important for the infusion perturbations (Figures 9b and S30–S34) since these extrapolations of the model result in depleted resting monocyte levels. Additional work remains to study the sensitivity of $k_{\mathrm{MR}}\;$ in these scenarios. Furthermore, if simulating a pathogenic insult, we suspect that the influence of this parameter on system dynamics would significantly increase to clear an infection of much greater magnitude than is safely observed in an endotoxin challenge.

In Figure 9, several parameter values were marked as outliers for continuous infusion and bolus subject‐specific model fits. These parameters correspond to subjects from both studies that exhibited abnormal cytokine responses for at least one of the measured cytokines. Given this, we hypothesize that these subjects may experience a more severe response or even enhanced complications to a clinical inflammation event. While this requires further investigation, it could be explored in silico by mathematical modelling.

We also explore variations of endotoxin infusion duration and total dose in Figure 10. A comparable simulation was conducted by Windoloski et al. (2023) on a bolus endotoxin model where the total dose was increased, representing the administration of more potent immune stimuli that cannot safely be given to humans and the stimuli strength of clinical infection. Both our study and Windoloski et al. (2023) observed enhanced cytokine production, but the current study showed a lack of significant dose response in cytokines except in IL‐10. Experimental studies such as Lipcsey et al. (2006) and Suffredini and Noveck (2014) studied the impact of endotoxin dose strength in pigs and humans, respectively, and both studies observed significant cytokine increases as the dose increased. However, these studies are not directly comparable to the current study due to differences in study population, endotoxin type, or administration method. We suggest that the weak dose response for the pro‐inflammatory cytokines in our study could be due to a slow monocyte regeneration rate, where the supply of resting monocytes is depleted too low from the initially larger insult and cannot activate enough monocytes to mount a comparable response. Since our study is hypothesis‐generating, examining the impact of monocyte regeneration could be a probable future direction to verify our results.

Our simulations extending the continuous infusion duration correspond to the clinical scenario of continual systemic aggravation by inflammatory stimuli. In this case, the model produces up to approximately 36 h of elevated immune markers depending on the length of the endotoxin infusion. It also displays attributes similar to endotoxin tolerance, a clinical phenomenon related to a reduced response to endotoxin after initial exposure (West & Heagy, 2002), through the appearance of multiple decreasing cytokine peaks when continual endotoxin administration is given across 20–32 h. Oscillations in cytokine concentrations also arise for 24 and 36‐h infusions when the total endotoxin dose is increased from 2 ng kg^−1^ to 4, 8 and 16 ng kg^−1^ (Figures S33 and S34 in Supporting Information), showing that the system can produce fluctuating behaviour for prolonged periods of inflammation if the stimuli are large enough. These cytokine oscillations that occur could also be clinically relevant with reference to recurrent infections where the system returns close to baseline before peaking again. In a clinical setting, however, the inflammatory stimulus is a live pathogen whose concentration would also fluctuate, compared to an endotoxin challenge where the administration of endotoxin is constant until cessation of infusion. This model of prolonged inflammation can be used to study inflammatory dynamics over longer periods and test or validate treatments for inflammatory conditions given the longer endotoxin exposure window.

### 4.1 Limitations

A primary limitation is that our model is calibrated to two data sets from Berg et al. (2012) and Janum et al. (2016). Although both administered a total dose of 2 ng kg^−1^ of endotoxin and had similar experimental protocols, the endotoxin was sourced from different vendors and was conducted on a different set of individuals. Thus, the studies are not statistically independent. While using data from two separate studies is a limitation, we accounted for this using modelling techniques for accurate comparison. Furthermore, while there are currently no dose–response comparisons for a 2 ng kg^−1^ endotoxin dose with these two sources, two studies have administered a 0.3 ng kg^−1^ bolus endotoxin dose using both sources. Taudorf et al. (2007) utilized Batch G2 B274 (The United States Pharmacopeial Convention, Inc., Rockville, MD, USA) and Andreasen et al. (2009) used Lot EC‐6 (US Pharmacopeial Convention). Both studies produced very similar cytokine peaks and dynamic timing. TNF‐α peaked at approximately 15 pgml^−1^ two hours after endotoxin administration, and IL‐6 peaked around 35 pgml^−1^ three hours after endotoxin administration. While these two endotoxin batches produced similar dynamics, utilizing additional endotoxin challenge data or, more ideally, comparing the immune responses during both endotoxin administration strategies on the same subjects using the same endotoxin batch would yield the best results. Our study prompts the need for future endotoxin studies using the same endotoxin batch in a randomized crossover design to measure the cytokine responses in the same population using the two different dose regimes.

Furthermore, there is widespread individual variation in the human immune response, evidenced by the individual subject data used in this study (Figures 2 and 3). However, this is not uncommon. It is well‐known that immune responses vary between individuals due in part to uncontrollable factors such as genetics, age, sex, seasonal and circadian influences, and environmental effects (Brodin & Davis, 2017). Additionally, only a few studies administer large endotoxin doses (such as 2 ng kg^−1^ as used here) as both a bolus and a continuous infusion. While Taudorf et al. (2007) administered 0.3 ng kg^−1^ of endotoxin as a bolus and a continuous infusion, the cytokine concentrations were notably lower than those from a larger dose (Janum et al., 2016; Krabbe et al., 2001) and near or below reported concentrations in septic patients (Berg et al., 2012; Casey et al., 1993; Wu et al., 2009). Torres et al. (2022) suggested that most patients are likely in the immunosuppressive stage of sepsis upon hospital admittance, so we suspect initial inflammation levels could be higher than reported in sepsis studies. Therefore, data from a 2 ng kg^−1^ endotoxin challenge likely yields more realistic cytokine concentrations than those observed in sepsis and should be used in our study.

Another limitation is that we do not have enough data to validate our endotoxin perturbation results on the continuous infusion model. Experimental data for a continuous infusion of larger endotoxin doses in humans is not seen in the literature except in Kiers et al. (2017) (who administers a total of 4 ng kg^−1^ of endotoxin) since larger doses of endotoxin are considered unsafe (Bahador & Cross, 2007). Safety may also play a role in the lack of experimental studies administering endotoxin for an extensive time beyond 4 h. There are animal studies such as that by Castegren et al. (2012) that conducted longer infusions, but contrast in the physiological makeup between animals (mice, pigs) and humans (Pabst, 2020) could result in different responses between species than is observed in our simulations. However, the observed oscillatory, recurring behaviour has been seen in humans during urinary tract infections, yeast infections and children's ear infections (Ballow, 2008; Hooton, 2001). Further model analysis and verification against experimental studies would be needed to determine whether this behaviour is a product of the model structure or a consequence of extended endotoxin infusions.

Although our mathematical model is highly nonlinear and complex, there are direct elements of the immune response (cells such as macrophages and neutrophils, cytokines such as IL‐1β and transforming growth factor β, and signalling pathways such as the nuclear factor κB pathway) and other sources of immune regulation (cardiovascular, nerve, hormonal, metabolic) that are not included here. Although Janum et al. (2016) reported that IL‐1β was measured in the bolus study, its concentrations were not detectable. Previous work (Dobreva et al., 2021) explored interactions of immune, cardiovascular, thermal and pain responses during a bolus endotoxin challenge, and Windoloski et al. (2023) expanded on that model to include hormonal regulation. Including these additional factors in the model dynamics could provide better insight into processes that activate at different speeds or strengths when the endotoxin challenge administration method is varied between a bolus and continuous infusion. A deeper understanding of continuous infusion dynamics could provide a better translational mathematical model of systemic inflammation, such as sepsis, encompassing multi‐organ dynamics.

### 4.2 Future work

Further investigation of this work would involve an additional examination of how initial conditions impact the model dynamics, which could include performing a multi‐objective optimization to determine the impact of fixed parameters on the different administration methods. We also plan to expand our study of continuous infusion dynamics to include immune interactions with other systems, such as thermal, pain and metabolic regulation and the cardiovascular and neuroendocrine systems, which builds upon the work in Windoloski et al. (2023). These components are well‐known to impact immune response and regulation (Dobreva et al., 2021; Hjemdahl et al., 2011; Janum et al., 2016; Kenney & Ganta, 2014; A. Miller et al., 2010; Varela et al., 2018), and studying how a continuous infusion affects these elements can enhance understanding of clinically prolonged inflammation events. Additionally, while we simulate longer infusions in this study, we do not have human experimental data to verify these predictions. Future directions could focus on replicating the pig study from Castegren et al. (2012) to investigate the dynamics of longer endotoxin infusions. Furthermore, while an endotoxin challenge attempts to mimic the dynamics of a clinical‐level immune insult, its duration is finite. It cannot simulate the extensive effects of an actual infection or trauma. Therefore, mathematical modelling can extrapolate dynamics from controlled environments to clinical relevance by looking at the impact of age, smoking, diabetes or cancer on immune responses. Additional future directions of this study would focus on transitioning our endotoxin immune response model to a model of sepsis, a life‐threatening condition involving hyperactive immune responses and subsequent organ failure that is still not fully understood (Nedeva et al., 2019). Much research has been focused on identifying a universal biomarker and treatment of sepsis, but one has yet to be accepted within the scientific community (Cecconi et al., 2018). However, recent progress has been made with the proposal of several candidates, including administering vitamin C to sepsis patients (Kashiouris et al., 2020; Wald et al., 2022). Adapting our current model to a model of sepsis could help improve our understanding of the mechanisms of sepsis and provide insight into the efficacy of potential sepsis treatments.

### 4.3 Conclusion

To enhance the understanding of potential mechanisms impacting immune responses to endotoxin, we devised a physiologically based mathematical model simulating mean and subject‐specific dynamics in human volunteers exposed to continuous and bolus endotoxin administration. Comparison of subject‐specific optimized parameter values revealed significant differences in the monocyte activation rate of IL‐10 and recovery rates of pro‐inflammatory cytokines TNF‐α and IL‐8. This suggests that increased IL‐10 activation by monocytes and slower recovery rates of pro‐inflammatory cytokines could play a role in the more pronounced anti‐inflammatory response and smaller secondary cytokine response seen in the continuous infusion data. Additionally, these factors likely influence the system's elongated and more gradual response to the endotoxin, as seen by the statistically significant later peak concentration times of all cytokines during the continuous infusion. Individuals with abnormal cytokine responses also reported statistically outlying optimal parameter values, suggesting their responses to a clinical infection could result in enhanced (outlying) reactions and complications. Simulations of the continuous infusion for a longer duration or increased dose amount display the model's capability to predict immune responses to prolonged inflammation or more potent inflammatory stimuli. Future directions of this work focus on verifying the hypothesized differing mechanisms between endotoxin administration methods through experimental crossover studies, development of a whole‐body response model to study the immune mechanisms occurring during a continuous infusion, and translation of this model to study clinically observed inflammation in sepsis patients.

## AUTHOR CONTRIBUTIONS

Kristen A. Windoloski, Ronan M. G. Berg and Mette S. Olufsen were responsible for the design of the study and the acquisition, analysis, or interpretation of data. Susanne Janum was responsible for the acquisition of data. Kristen A. Windoloski and Mette S. Olufsen were responsible for the mathematical model calibration, simulation and interpretations. Kristen A. Windoloski, Susanne Janum, Ronan M. G. Berg and Mette S. Olufsen were responsible for drafting or critically editing the manuscript. All authors have read and approved the final version of this manuscript and agree to be accountable for all aspects of the work in ensuring that questions related to the accuracy or integrity of any part of the work are appropriately investigated and resolved. All persons designated as authors qualify for authorship, and all those who qualify for authorship are listed.

## CONFLICT OF INTEREST

The authors declare no conflicts of interest.

## Supporting information

Mathematical equations and analysis (scaling analysis for nominal parameters, sensitivity analysis, subset selection and statistical methods), model plots for the subject‐specific optimizations, additional endotoxin perturbation simulations and Bayesian measures of uncertainty are included in a supplemental document for further reading.

## Data Availability

This study utilizes cytokine data from two previously published studies Berg et al. (2012) and Janum et al. (2016). The MATLAB code for computer simulations for the mean and each subject in both studies is available on the GitHub repository: https://github.com/msolufse/Inflammation_Bolus_Cont. The experimental data underlying our findings can be shared upon reasonable request and directed to the corresponding author.

## 1

Table A1, Table A2.

**TABLE A1:** Parameter descriptions, units, and nominal values for the continuous infusion (C) and bolus injection (B) endotoxin models.

Parameter	Description	Unit	Nominal value (C)	Nominal value (B)
Endotoxin (E)				
*D*_h_	E administered per hour	ng kg^−1^ h^−1^	0.5	0.0
*D*_ad_	E administered duration	h	4	0
*k*_E_	E decay rate	h^−1^	1.01	1.01
Monocytes (M)				
*k*_MR_	Regeneration rate	h^−1^	0.006	0.006
*k*_MA_	Activated decay rate	h^−1^	2.51	2.51
*k*_MTNF_	Activation rate by TNF	h^−1^	9.00	8.65
*k*_M_	Activation rate by E	h^−1^	0.041	0.041
η_ME_	Upregulation half max of E	ng kg^−1^	2.0	3.3
η_M10_	Downregulation half max of IL10	pg ml^−1^	4.39	3.88
η_MTNF_	Upregulation half max of TNF	pg ml^−1^	222	141
*h*_ME_	Upregulation exp of E on M	non dim	1	1
*h*_M10_	Upregulation exp of IL10 on M	non dim	0.3	0.3
*h*_MTNF_	Upregulation exp of TNF on M	non dim	3.16	3.16
*M*_∞_	Carrying capacity	noc	30,000	30,000
TNF‐α (TNF)				
*k*_TNF_	Decay rate	h^−1^	0.6	1
*k*_TNFM_	Activation rate by M	pg ml^−1^ h^−1^ noc^−1^	1.333	0.845
η_TNF10_	Downregulation half max of IL10	pg ml^−1^	17.6	15.5
η_TNF6_	Downregulation half max of IL6	pg ml^−1^	560	560
*h*_TNF10_	Downregulation exp of IL10 on *TNF*	non dim	3	3
*h*_TNF6_	Upregulation exp of IL6 on TNF	non dim	2	2
*w*_TNF_	Baseline concentration	pg ml^−1^	13.9	8.79
IL‐6 (IL6)				
*k*_6_	Decay rate	h^−1^	0.66	0.66
*k*_6M_	Activation rate by M	pg ml^−1^ h^−1^ noc^−1^	0.81	0.81
*K*_6TNF_	Activation rate by TNF	pg ml^−1^ h^−1^ noc^−1^	0.81	0.81
η_610_	Downregulation half max of IL10	pg ml^−1^	35.2	31.1
η_66_	Downregulation half max of IL6	pg ml^−11^	560	560
η_6TNF_	Upregulation half max of TNF	pg ml^−1^	411	261
*h*_610_	Downregulation exp of IL10 on IL6	non dim	1	4
*h*_66_	Downregulation exp of IL6 on IL6	non dim	1	1
*h*_6TNF_	Downregulation exp of TNF on IL6	non dim	2	2
*w*_6_	Baseline concentration	pg ml^−1^	0.61	0.61
IL‐8 (IL8)				
*k*_8_	Decay rate	h^−1^	0.66	0.66
*k*_8M_	Activation rate by M	pg ml^−1^ h^−1^ noc^−1^	0.509	0.789
*k*_8TNF_	Activation rate by TNF	pg ml^−1^ h^−1^ noc^−1^	0.509	0.789
η_810_	Downregulation half max of IL10	pg ml^−1^	17.6	15.5
η_8TNF_	Upregulation half max of TNF	pg ml^−1^	411	261
*h*_810_	Downregulation exp of IL10 on IL8	non dim	1.5	1.5
*h*_8TNF_	Upregulation exp of TNF on IL8	non dim	3	3
*w*_8_	Baseline concentration	pg ml^−1^	2.70	4.18
IL‐10 (IL10)				
*k*_10_	Decay rate	h^−1^	0.4	0.8
*k*_10M_	Activation rate by M	pg ml^−1^ h^−1^ noc^−1^	0.019	0.017
*k*_106_	Activation rate by IL6	pg ml^−1^ h^−1^ noc^−1^	0.019	0.017
η_106_	Upregulation half max of IL6	pg ml^−1^	560	560
*h*_106_	Upregulation exp of IL6 on IL10	non dim	3.68	3.68
*w*_10_	Baseline concentration	pg ml^−1^	4.24	3.75

**TABLE A2:** Subject‐specific parameter values as mean (SD) and estimated parameter *P*‐values with significance level α = 0.05.

Parameter	Continuous infusion mean (SD)	Bolus mean (SD)	*P*‐value
*k*_MA_	3.88 (1.06)	3.59 (0.587)	*P* = 0.465 (*m* = 9,*n* = 20)
η_M10_	5.69 (3.38)	5.72 (2.79)	
η_MTNF_	249 (152)	152 (114)	
*k*_TNF_	**0.642 (0.274)**	**1.78 (0.406)**	*P*< 0.0001 (*m* = 9,*n* = 19)
*k*_TNFM_	**2.25 (1.56)**	**1.44 (0.948)**	*P* = 0.106 (*m* = 9,*n* = 19)
η_TNF10_	22.8 (13.5)	22.9 (11.2)	
η_TNF6_	674 (356)	686 (346)	
*w*_TNF_	7.22 (5.09)	6.86 (5.22)	
*k*_6M_	0.975 (0.516)	0.992 (0.500)	
*k*_6TNF_	0.975 (0.516)	0.992 (0.500)	
η_610_	45.5 (27.1)	45.8 (22.3)	
η_66_	674 (356)	686 (346)	
η_6TNF_	461 (280)	280 (211)	
*w*_6_	0.658 (0.484)	0.831 (0.690)	
***k*_8_**	**0.439 (0.127)**	**0.719 (0.174)**	*P*<0.0001 (*m* = 8,*n* = 9)
***k*_8M_**	**0.733 (0.331)**	**0.941 (0.386)**	*P* = 0.0615 (*m* = 8,*n* = 19)
*k*_8TNF_	0.542 (0.202)	0.884 (0.154)	
η_810_	22.8 (13.5)	22.9 (11.2)	
η_8TNF_	461 (280)	280 (211)	
*w*_8_	5.03 (5.73)	3.33 (1.07)	
***k*_10M_**	**0.0518 (0.0339)**	**0.0238 (0.012)**	*P* = 0.0142 (*m* = 7,*n* = 19)
*k*_106_	0.0250 (0.0148)	0.0251 (0.012)	
η_106_	674 (356)	686 (346)	
*w*_10_	5.82 (5.04)	6.280 (3.24)	

*Note*: Estimated parameters are marked in bold and remaining parameters were scaled from their nominal values. Mean (SD) values are computed using subject parameter values, including abnormal responses. *P*‐values were calculated by removing the abnormal responses before hypothesis testing, with *m* subjects from the continuous infusion and *n* subjects from the bolus study being included.

## References

[eph13501-bib-0001] Akimoto, H. , Yamada, A. , & Takikawa, O. (2007). Up‐regulation of the brain indoleamine 2,3‐dioxygenase activity in a mouse model of alzheimer's disease by systemic endotoxin challenge. International Congress Series, 1304, 357–361.

[eph13501-bib-0002] Andreasen, A. S. , Pedersen‐Skovsgaard, T. , Mortensen, O. H. , van Hall, G. , Moseley, P. L. , & Pedersen, B. K. (2009). The effect of glutamine infusion on the inflammatory response and hsp70 during human experimental endotoxaemia. Critical Care, 13(1), 1–8. 10.1186/cc7696 PMC2688119 19173710

[eph13501-bib-0003] Bahador, M. , & Cross, A. (2007). Review: From therapy to experimental model: A hundred years of endotoxin administration to human subjects. Endotoxin Research, 13(5), 251–279. 10.1177/0968051907085986 17986486

[eph13501-bib-0004] Ballow, M. (2008). Approach to the patient with recurrent infections. Clinical Reviews in Allergy & Immunology, 34(2), 129–140. 18330724 10.1007/s12016-007-8041-2

[eph13501-bib-0005] Bangsgaard, E. , Hjorth, P. , Olufsen, M. S. , Mehlsen, J. , & Ottesen, J. T. (2017). Integrated inflammatory stress (itis) model. Bulletin of Mathematical Biology, 79(7), 1487–1509. 28643132 10.1007/s11538-017-0293-2

[eph13501-bib-0006] Banks, H. , Davidian, M. , Samuels, J. , & Sutton, K. (2009). An inverse problem statistical methodology summary. In G. Chowell , J. Hyman , L. Bettencourt , & C. Castillo‐Chavez (Eds), Mathematical and statistical estimation approaches in epidemiology (pp. 249–303). Springer Amsterdam.

[eph13501-bib-0007] Bemelmans, M. H. , van Tits, L. J. , & Buurman, W. A. (1996). Tumor necrosis factor: Function, release and clearance. Critical Reviews in Immunology, 16(1), 1–11. 8809470 10.1615/critrevimmunol.v16.i1.10

[eph13501-bib-0008] Berg, R. , Plovsing, R. , Ronit, A. , Bailey, D. , Holstein‐Rathlou, N. , & Møller, K. (2012). Disassociation of static and dynamic cerebral autoregulatory performance in healthy volunteers after lipopolysaccharide infusion and in patients with sepsis. American Journal of Physiology, 303(11), R1127–R1135. 23076874 10.1152/ajpregu.00242.2012

[eph13501-bib-0009] Biancotto, A. , Wank, A. , Perl, S. , Cook, W. , Olnes, M. J. , Dagur, P. K. , Fuchs, J. C. , Langweiler, M. , Wang, E. , & McCoy, J. P. (2013). Baseline levels and temporal stability of 27 multiplexed serum cytokine concentrations in healthy subjects. PLoS ONE, 8(12), e76091. 24348989 10.1371/journal.pone.0076091 PMC3861126

[eph13501-bib-0010] Bickel, M. (1993). The role of interleukin‐8 in inflammation and mechanisms of regulation. Journal of Periodontology, 64, (5 Suppl), 456–460. 8315568

[eph13501-bib-0011] Brady, R. (2017). Mathematical modeling of the acute inflammatory response & cardiovascular dynamics in young men. In *Mathematics*. Ph.D. North Carolina State University.

[eph13501-bib-0012] Brady, R. , Frank‐Ito, D. , Tran, H. , Janum, S. , Møller, K. , Brix, S. , Ottesen, J. T. , Mehlsen, J. , & Olufsen, M. S. (2018). Personalized mathematical model of endotoxin‐induced inflammatory responses in young men and associated changes in heart rate variability. Mathematical Modelling of Natural Phenomena, 13(5), 42.

[eph13501-bib-0013] Brodin, P. , & Davis, M. (2017). Human immune system variation. Nature Reviews Immunology, 17, 21–29. 10.1038/nri.2016.125 PMC5328245 27916977

[eph13501-bib-0014] Burnham, K. , & Anderson, D. (2002). Model selection and multimodal inference: A practical information‐theoretic approach. Springer.

[eph13501-bib-0015] Casey, L. C. , Balk, R. A. , & Bone, R. C. (1993). Plasma cytokine and endotoxin levels correlate with survival in patients with the sepsis syndrome. Annals of Internal Medicine, 119(8), 771–778. 8379598 10.7326/0003-4819-119-8-199310150-00001

[eph13501-bib-0016] Castegren, M. , Lipcsey, M. , Söderberg, E. , Skorup, P. , Eriksson, M. , Larsson, A. , & Sjölin, J. (2012). Differences in organ dysfunction in endotoxin‐tolerant pigs under intensive care exposed to a second hit of endotoxin. Shock, 37(5), 501–510. 22266970 10.1097/SHK.0b013e318249bb0d

[eph13501-bib-0017] Cecconi, M. , Evans, L. , Levy, M. , & Rhodes, A. (2018). Sepsis and septic shock. The Lancet, 392, 75–87. 10.1016/S0140-6736(18)30696-2 29937192

[eph13501-bib-0018] Chow, C. C. , Clermont, G. , Kumar, R. , Lagoa, C. , Tawadrous, Z. , Gallo, D. , Betten, B. , Bartels, J. C. , Constantine, G. , Fink, M. P. , Billiar, T. R. , & Vodovotz, Y. (2005). The acute inflammatory response in diverse shock states. Shock, 24(1), 74–84. 15988324 10.1097/01.shk.0000168526.97716.f3

[eph13501-bib-0019] Clodi, M. , Vila, G. , Geyeregger, R. , Riedl, M. , Stulnig, T. , Struck, J. , Luger, T. , & Luger, A. (2008). Oxytocin alleviates the neuroendocrine and cytokine response to bacterial endotoxin in healthy men. American Journal of Physiology‐Endocrinology and Metabolism, 295(3), E686–E691. 18593851 10.1152/ajpendo.90263.2008

[eph13501-bib-0020] Copeland, S. , Warren, H. , Lowry, S. , Calvano, S. , & Remick, D. (2005). Clinical inflammatory response to endotoxin in mice and humans. Clinical and Diagnostic Laboratory Immunology, 12, 60–67. 15642986 10.1128/CDLI.12.1.60-67.2005 PMC540200

[eph13501-bib-0021] Cunningham, C. , Wilcockson, D. , Campion, S. , Lunnon, K. , & Perry, V. (2005). Central and systemic endotoxin challenges exacerbate the local inflammatory response and increase neuronal death during chronic neurodegeneration. Journal of Neuroscience, 25(40), 9275–9284. 16207887 10.1523/JNEUROSCI.2614-05.2005 PMC6725757

[eph13501-bib-0022] Danz, A. (2023). boxplotgroup. MATLAB Central File Exchange.

[eph13501-bib-0023] Day, J. , Rubin, J. , Vodovotz, Y. , Chow, C. , Reynolds, A. , & Clermont, G. (2006). A reduced mathematical model of the acute inflammatory response: II. Capturing scenarios of repeated endotoxin administration. Journal of Theoretical Biology, 242(1), 237–256. 16616206 10.1016/j.jtbi.2006.02.015

[eph13501-bib-0025] Dobreva, A. , Brady‐Nicholls, R. , Larripa, K. , Puelz, C. , Mehlsen, J. , & Olufsen, M. (2021). A physiological model of the inflammatory‐thermal‐pain‐cardiovascular interactions during an endotoxin challenge. The Journal of Physiology, 599(5), 1459–1485. 33450068 10.1113/JP280883

[eph13501-bib-0026] Dodge, Y. (2008). The concise encyclopedia of statistics. Springer.

[eph13501-bib-0027] Easson, A. , Bode, B. , Fischer, C. , & Souba, W. (1998). Effects of endotoxin challenge on hepatic amino acid transport during cancer. Journal of Surgical Research, 77(1), 29–34. 9698528 10.1006/jsre.1998.5323

[eph13501-bib-0028] Fitzal, F. , DeLano, F. , Young, C. , Rosario, H. , Junger, W. G. , & Schmid‐Schönbein, G. (2003). Pancreatic enzymes sustain systemic inflammation after an initial endotoxin challenge. Surgery, 134(3), 446–456. 14555932 10.1067/s0039-6060(03)00168-5

[eph13501-bib-0029] Foteinou, P. , Calvano, S. , Lowry, S. , & Androulakis, I. (2009). Modeling endotoxin‐induced systemic inflammation using an indirect response approach. Mathematical Biosciences, 217(1), 27–42. 18840451 10.1016/j.mbs.2008.09.003 PMC3045970

[eph13501-bib-0030] Foteinou, P. , Calvano, S. , Lowry, S. , & Androulakis, I. (2011). A physiological model for autonomic heart rate regulation in human endotoxemia. Shock, 35(3), 229–239. 21063241 10.1097/SHK.0b013e318200032b PMC3045969

[eph13501-bib-0031] Fredriksson, K. , Fläring, U. , Guillet, C. , Wernerman, J. , & Rooyackers, O. (2009). Muscle mitochondrial activity increases rapidly after an endotoxin challenge in human volunteers. Acta Anaesthesiologica Scandinavica, 53(3), 299–304. 19243315 10.1111/j.1399-6576.2008.01851.x

[eph13501-bib-0032] Givalois, L. , Dornand, J. , Mekaouche, M. , Solier, M. , Bristow, A. , Ixart, G. , Siaud, P. , Assenmacher, I. , & Barbanel, G. (1994). Temporal cascade of plasma level surges in ACTH, corticosterone, and cytokines in endotoxin‐challenged rats. American Journal of Physiology, 267(1 Pt 2), R164–R170. 8048620 10.1152/ajpregu.1994.267.1.R164

[eph13501-bib-0033] Heine, H. , Rietschel, E. T. , & Ulmer, A. (2001). The biology of endotoxin. Molecular Biotechnology, 19(3), 279–296. 11721624 10.1385/MB:19:3:279

[eph13501-bib-0034] Hjemdahl, P. , Rosengren, A. , & Steptoe, A. (2011). Stress and cardiovascular disease. Springer.

[eph13501-bib-0035] Ho, T. , Clermont, G. , & Parker, R. (2013). A model of neutrophil dynamics in response to inflammatory and cancer chemotherapy challenges. Computers & Chemical Engineering, 51, 187–196.

[eph13501-bib-0036] Hooton, T. M. (2001). Recurrent urinary tract infection in women. International Journal of Antimicrobial Agents, 17, 259–268. 11295405 10.1016/s0924-8579(00)00350-2

[eph13501-bib-0037] Janum, S. , Nielsen, S. , Werner, M. , Mehlsen, J. , Kehlet, H. , & Møller, K. (2016). Pain perception in healthy volunteers: Effect of repeated exposure to experimental systemic inflammation. Innate Immunity, 22(7), 546–556. 27554053 10.1177/1753425916663638

[eph13501-bib-0038] Jin, J. , Han, X. , & Yu, Q. (2013). Interleukin‐6 induces the generation of IL‐10‐producing TR1 cells and suppresses autoimmune tissue inflammation. Journal of Autoimmunity, 40, 28–44. 22921334 10.1016/j.jaut.2012.07.009 PMC3524403

[eph13501-bib-0039] Johnston, G. , & Webster, N. (2009). Cytokines and the immunomodulatory function of the vagus nerve. British Journal of Anaesthesia, 102(4), 453–462. 19258380 10.1093/bja/aep037

[eph13501-bib-0040] Kashiouris, M. , L'heureux, M. , Cable, C. , Fisher, B. , Leichtle, S. , & Fowler, A. (2020). The emerging role of vitamin C as a treatment for sepsis. Nutrients, 12(2), 1–16. 10.3390/nu12020292 PMC7070236 31978969

[eph13501-bib-0041] Kenney, M. , & Ganta, C. (2014). Autonomic nervous system and immune system interactions. Comprehensive Physiology, 4(4), 1177. 24944034 10.1002/cphy.c130051 PMC4374437

[eph13501-bib-0042] Kiers, D. , Koch, R. , Hamers, L. , Gerretsen, J. , Thijs, E. , Van Ede, L. , Riksen, N. , Kox, M. , & Pickkers, P. (2017). Characterization of a model of systemic inflammation in humans in vivo elicited by continuous infusion of endotoxin. Scientific Reports, 7, 1–10. 28054645 10.1038/srep40149 PMC5215288

[eph13501-bib-0043] Krabbe, K. , Bruunsgaard, H. , Hansen, C. , Møller, K. , Fonsmark, L. , Qvist, J. , Madsen, P. , Kronborg, G. , Andersen, H. , Skinhøj, P. , & Pedersen, B. (2001). Ageing is associated with a prolonged fever response in human endotoxemia. Clinical and Diagnostic Laboratory Immunology, 8(2), 33–338. 10.1128/CDLI.8.2.333-338.2001 PMC96058 11238217

[eph13501-bib-0044] Kucharzik, T. , Lügering, N. , Pauels, H. , Domschke, W. , & Stoll, R. (1998). IL‐4, IL‐10 and IL‐13 down‐regulate monocyte‐chemoattracting protein‐1 (MCP‐1) production in activated intestinal epithelial cells. Clinical and Experimental Immunology, 111(1), 152–157. 9472675 10.1046/j.1365-2249.1998.00481.x PMC1904856

[eph13501-bib-0045] Kumar, R. , Clermont, G. , Vodovotz, Y. , & Chow, C. C. (2004). The dynamics of acute inflammation. Journal of Theoretical Biology, 230(2), 145–155. 15321710 10.1016/j.jtbi.2004.04.044

[eph13501-bib-0046] Leijte, G. , Kiers, D. , Van der Heijden, W. , Jansen, A. , Gerretsen, J. , Boerrigter, V. , Netea, M. , Kox, M. , & Pickkers, P. (2019). Treatment with acetylsalicylic acid reverses endotoxin tolerance in humans in vivo: A randomized placebo‐controlled study. Critical Care Medicine, 47(4), 508–516. 30585832 10.1097/CCM.0000000000003630 PMC6426341

[eph13501-bib-0047] Lipcsey, M. , Larsson, A. , Eriksson, M. B. , & Sjölin, J. (2006). Inflammatory, coagulatory and circulatory responses to logarithmic increases in the endotoxin dose in the anaesthetised pig. Journal of Endotoxin Research, 12(2), 99–112. 16690013 10.1179/096805106X89053

[eph13501-bib-0048] Lorenz, W. , Buhrmann, C. , Mobasheri, A. , Lueders, C. , & Shakibaei, M. (2013). Bacterial lipopolysaccharides form procollagen‐endotoxin complexes that trigger cartilage inflammation and degeneration: Implications for the development of rheumatoid arthritis. Arthritis Research & Therapy, 15(5), R111. 24020912 10.1186/ar4291 PMC3978890

[eph13501-bib-0049] Malek, H. , Ebadzadeh, M. , Safabakhsh, R. , Razavi, A. , & Zaringhalam, J. (2015). Dynamics of the HPA axis and inflammatory cytokines: Insights from mathematical modeling. Computers in Biology and Medicine, 67, 1–12. 26476562 10.1016/j.compbiomed.2015.09.018

[eph13501-bib-0050] Mathison, J. C. , & Ulevitch, R. J. (1979). The clearance, tissue distribution, and cellular localization of intravenously injected lipopolysaccharide in rabbits. Journal of Immunology Research, 123(5), 2133–2143. 489976

[eph13501-bib-0051] Matsushima, K. , Yang, D. , & Oppenheim, J. J. (2022). Interleukin‐8: An evolving chemokine. Cytokine, 153, 155828. 35247648 10.1016/j.cyto.2022.155828

[eph13501-bib-0052] Merrill, J. , You, J. , Constable, C. , Leeman, S. , & Amar, S. (2011). Whole‐body deletion of lps‐induced TNF‐α factor (litaf) markedly improves experimental endotoxic shock and inflammatory arthritis. The Proceedings of the National Academy of Sciences, 108(52), 21247–21252. 10.1073/pnas.1111492108 PMC3248491 22160695

[eph13501-bib-0053] Miao, H. , Xia, X. , Perelson, A. , & Wu, H. (2011). On identifiability of nonlinear ode models and applications in viral dynamics. Siam Review, 53(1), 3–39. 21785515 10.1137/090757009 PMC3140286

[eph13501-bib-0054] Miller, A. , Pearce, B. , Ruzek, M. , & Biron, C. (2010). Interactions between the hypothalamic pituitary‐adrenal axis and immune system during viral infection: Pathways for environmental effects on disease expression. In B. S. Mcewen , & H. M. Goodman (Eds), *Handbook of physiology*, *section 7. The endocrine system. Volume IV Coping with the environment: Neural and endocrine mechanisms* (vol. Supplement 23. pp. 425–450). Oxford University Press.

[eph13501-bib-0055] Miller, M. , Samuelson, C. , Hiramoto, R. , & Ward, J. (1979). Endotoxin toxicity in rats with 6‐sulfanilamidoindazole arthritis. Infection and Immunity, 25(1), 337–344. 383619 10.1128/iai.25.1.337-344.1979 PMC414457

[eph13501-bib-0056] Murphy, K. (2012). Janeway's Immunobiology. Garland Science, London, UK.

[eph13501-bib-0057] Nedeva, C. , Menassa, J. , & Puthalakath, H. (2019). Sepsis: Inflammation is a necessary evil. Frontiers in Cell and Developmental Biology, 7, 108. 31281814 10.3389/fcell.2019.00108 PMC6596337

[eph13501-bib-0058] Olufsen, M. S. , & Ottesen, J. T. (2013). Patient specific parameter estimation and heart rate regulation. Journal of Mathematical Biology, 67(1), 39–68. 22588357 10.1007/s00285-012-0535-8 PMC3526689

[eph13501-bib-0059] Pabst, R. (2020). The pig as a model for immunology research. Cell and Tissue Research, 380(2), 287–304. 32356014 10.1007/s00441-020-03206-9 PMC7223737

[eph13501-bib-0060] Parker, R. , Hogg, J. , Roy, A. , Kellum, J. , Rimmelé, T. , Daun‐Gruhn, S. , Fedorchak, M. , Valenti, I. , Federspiel, W. , Rubin, J. , Vodovotz, Y. , Lagoa, C. , & Clermont, G. (2016). Modeling and hemofiltration treatment of acute inflammation. Processes, 4(4), 1–33. 10.3390/pr4040038 PMC7596788 33134139

[eph13501-bib-0061] Pope, S. , Ellwein, L. , Zapata, C. V. N. , Kelley, C. , & Olufsen, M. S. (2009). Estimation and identification of parameters in a lumped cerebrovascular model. Mathematical Biosciences and Engineering, 6(1), 93–115. 19292510 10.3934/mbe.2009.6.93

[eph13501-bib-0062] Rennen, H. J. , Boerman, O. C. , Oyen, W. J. , & Corstens, F. H. (2003). Kinetics of 99mtc‐labeled interleukin‐8 in experimental inflammation and infection. Journal of Nuclear Medicine, 44(9), 1502–1509. 12960199

[eph13501-bib-0063] Reynolds, A. , Rubin, J. , Clermont, G. , Day, J. , Vodovotz, Y. , & Ermentrout, B. (2006). A reduced mathematical model of the acute inflammatory response. I. Derivation of model and analysis of anti‐inflammation. Journal of Theoretical Biology, 242(1), 220–236. 16584750 10.1016/j.jtbi.2006.02.016

[eph13501-bib-0064] Rossol, M. , Heine, H. , Meusch, U. , Quandt, D. , Klein, C. , Sweet, M. , & Hauschildt, S. (2011). LPS‐induced cytokine production in human monocytes and macrophages. Critical Reviews in Immunology, 31(5), 379–446. 22142165 10.1615/critrevimmunol.v31.i5.20

[eph13501-bib-0065] Roy, A. , Daun, S. , Clermont, G. , Rubin, J. , Vodovotz, Y. , Lagoa, C. , & Parker, R. (2007). A mathematical model of acute inflammatory response to endotoxin challenge. In AIChE Annual Meeting , pp. 538, Salt Lake City, Utah.

[eph13501-bib-0066] Scheff, J. , Calvano, S. , Lowry, S. , & Androulakis, I. (2010). Modeling the influence of circadian rhythms on the acute inflammatory response. Journal of Theoretical Biology, 264(3), 1068–1076. 20307551 10.1016/j.jtbi.2010.03.026

[eph13501-bib-0067] Schwarz, G. (1978). Estimating the dimension of a model. Annals of Statistics, 6(2), 461–464.

[eph13501-bib-0068] Seber, G. , & Wild, C. (2003). Nonlinear regression. Wiley.

[eph13501-bib-0069] Shinozaki, S. , Inoue, Y. , Yang, W. , Fukaya, M. , Carter, E. , Ming‐Yu, Y. , Fischman, A. , Tompkins, R. , & Kaneki, M. (2010). Farnesyltransferase inhibitor improved survival following endotoxin challenge in mice. Biochemical and Biophysical Research Communications, 391(3), 1459–1464. 20034462 10.1016/j.bbrc.2009.12.094 PMC2813732

[eph13501-bib-0070] Sly, L. , Krzesicki, R. , Brashler, J. , Buhl, A. , McKinley, D. , Carter, D. , & Chin, J. (2001). Endogenous brain cytokine mRNA and inflammatory responses to lipopolysaccharide are elevated in the tg2576 transgenic mouse model of Alzheimer's disease. Brain Research Bulletin, 56(6), 581–588. 11786245 10.1016/s0361-9230(01)00730-4

[eph13501-bib-0071] Smith, R. C. (2014). *Uncertainty quantification*, *theory*, *implementation*, *and applications* . SIAM.

[eph13501-bib-0072] Su, B. , Zhou, W. , Dorman, K. , & Jones, D. (2009). Mathematical modelling of immune response in tissues. Computational and Mathematical Methods in Medicine, 10(1), 9–38.

[eph13501-bib-0073] Suffredini, A. , & Noveck, R. (2014). Human endotoxin administration as an experimental model in drug development. Clinical Pharmacology & Therapeutics, 96(4), 418–422. 25236665 10.1038/clpt.2014.146

[eph13501-bib-0074] Taudorf, S. , Krabbe, K. , Berg, R. , Møller, K. , Pedersen, B. , & Bruunsgaard, H. (2008). Common studied polymorphisms do not affect plasma cytokine levels upon endotoxin exposure in humans. Clinical and Experimental Immunology, 152(1), 147–152. 18307517 10.1111/j.1365-2249.2008.03612.x PMC2384055

[eph13501-bib-0075] Taudorf, S. , Krabbe, K. , Berg, R. , Pedersen, B. , & Møller, K. (2007). Human models of low grade inflammation: Bolus versus continuous infusion of endotoxin. Clinical and Vaccine Immunology: CVI, 14(3), 250–255. 17267590 10.1128/CVI.00380-06 PMC1828854

[eph13501-bib-0076] Tilg, H. , Trehu, E. , Atkins, M. , Dinarello, C. , & Mier, J. (1994). Interleukin‐6 (IL‐6) as an anti‐inflammatory cytokine: Induction of circulating IL‐1 receptor antagonist and soluble tumor necrosis factor receptor p55. Blood, 83(1), 113–118. 8274730

[eph13501-bib-0077] Torres, M. , Wang, J. , Yannie, P. , Ghosh, S. , Segal, R. , & Reynolds, A. (2019). Identifying important parameters in the inflammatory process with a mathematical model of immune cell influx and macrophage polarization. PLoS Computational Biology, 15(7), e1007172. 31365522 10.1371/journal.pcbi.1007172 PMC6690555

[eph13501-bib-0078] Torres, P. , Pickkers, P. , & van der Poll, T. (2022). Sepsis‐induced immunosuppression. Annual Review of Physiology, 84, 157–181. 10.1146/annurev-physiol-061121-040214 34705481

[eph13501-bib-0079] van Lier, D. , Geven, C. , Leijte, G. , & Pickkers, P. (2019). Experimental human endotoxemia as a model of systemic inflammation. Biochimie, 159, 99–106. 29936295 10.1016/j.biochi.2018.06.014

[eph13501-bib-0080] Varela, M. , Mogildea, M. , Moreno, I. , & Lopes, A. (2018). Acute inflammation and metabolism. Inflammation, 41(4), 1115–1127. 29404872 10.1007/s10753-018-0739-1

[eph13501-bib-0081] Verboogen, D. R. , Revelo, N. H. , Ter Beest, M. , & van den Bogaart, G. (2019). Interleukin‐6 secretion is limited by self‐signaling in endosomes. Journal of Molecular Cell Biology, 11(2), 144–157. 30016456 10.1093/jmcb/mjy038 PMC6392102

[eph13501-bib-0082] Wald, E. , Badke, C. , Hintz, L. , Spewak, M. , & Sanchez‐Pinto, L. (2022). Vitamin therapy in sepsis. Pediatric Research, 91(2), 328–336. 34333556 10.1038/s41390-021-01673-6 PMC8325544

[eph13501-bib-0083] West, M. A. , & Heagy, W. (2002). Endotoxin tolerance: A review. Critical Care Medicine, 30, (1 Suppl), S64–S73. 11782563

[eph13501-bib-0084] Windoloski, K. , Bangsgaard, E. , Dobreva, A. , Ottesen, J. , & Olufsen, M. (2023). A unified computational model for the human response to lipopolysaccharide‐induced inflammation. In B. Booß‐Bavnbek , J. H. Christensen , K. Richardson , & O. V. Codina (Eds), Multiplicity of time scales in complex systems. Springer.

[eph13501-bib-0085] Windoloski, K. (2023). Mathematical modeling of experimentally induced inflamation and sepsis. In *Mathematics*. Ph.D. North Carolina State University.

[eph13501-bib-0086] Wu, H.‐P. , Chen, C.‐K. , Chung, K. , Tseng, J.‐C. , Hua, C.‐C. , Liu, Y.‐C. , Chuang, D.‐Y. , & Yang, C.‐H. (2009). Serial cytokine levels in patients with severe sepsis. Inflammation Research, 58(7), 385–393. 19262987 10.1007/s00011-009-0003-0

[eph13501-bib-0087] Yassine, F. (2016). Effect of endotoxin challenge on normal, tumor‐initiated, and invasive human breast cells. Am University of Beirut.

[eph13501-bib-0088] Zamyatina, A. , & Heine, H. (2020). Lipopolysaccharide recognition in the crossroads of tlr4 and caspase‐4/11 mediated inflammatory pathways. Frontiers in Immunology, 11, 585146. 33329561 10.3389/fimmu.2020.585146 PMC7732686

[eph13501-bib-0089] Zeilhofer, H. U. , & Schorr, W. (2000). Role of interleukin‐8 in neutrophil signaling. Current Opinion in Hematology, 7, 178–182. 10786656 10.1097/00062752-200005000-00009

[eph13501-bib-0090] Zhang, Y.‐Y. , & Ning, B.‐T. (2021). Signaling pathways and intervention therapies in sepsis. Signal Transduction and Targeted Therapy, 6(1), 407. 34824200 10.1038/s41392-021-00816-9 PMC8613465

[eph13501-bib-0091] Zuckerman, S. , Ahmari, S. , Bryan‐Poole, N. , Evans, G. , Short, L. , & Glasebrook, A. (1996). Estriol: A potent regulator of TNF and IL‐6 expression in a murine model of endotoxemia. Inflammation, 20(6), 581–597. 8979148 10.1007/BF01488797

