# Supplementary material for: Characterization of differences in immune responses during bolus and continuous infusion endotoxin challenges using mathematical modelling
Source: Exp Physiol. 2024 Mar 11;109(5):689–710. doi: 10.1113/EP091552 (PMC11061636; doi:10.1113/EP091552)
Supplement: Supplementary file 1 — Mathematical equations and analysis (scaling analysis for nominal parameters, sensitivity analysis, subset selection and statistical methods), model plots for the subject‐specific optimizations, additional endotoxin perturbation simulations and Bayesian measures of uncertainty are included in a supplemental document for further reading. [file EPH-109-689-s001.pdf]

Characterization of differences in immune responses during bolus and continuous infusion endotoxin challenges using mathematical modeling: Supporting Information

Kristen A. Windoloski[1], Susanne Janum[2,4], Ronan M.G. Berg[3,4,5,6], and Mette S. Olufsen[1]

[1]Department of Mathematics, North Carolina State University, Raleigh, North Carolina, USA
[2]Frederiksberg and Bispebjerg Hospitals, Frederiksberg, Denmark
[3]Department of Clinical Physiology and Nuclear Medicine, Copenhagen University Hospital, Denmark
[4]Centre for Physical Activity Research, Rigshospitalet, Denmark
[5]Department of Biomedical Sciences, University of Copenhagen, Denmark
[6]Neurovascular Research Laboratory, University of South Wales, Pontypridd, UK

S1 Introduction

This document includes supporting information for the manuscript “Characterization of differences in immune responses during bolus and continuous infusion endotoxin challenges using mathematical modeling". In Section 2, we provide the mathematical and statistical techniques used to formulate and analyze the model. This includes detailing the scaling procedure used to determine nominal parameters, sensitivity analysis and subset selection methods used to generate a parameter subset to estimate, and statistical methods used to evaluate model fit and compute uncertainty bounds as reported in the main manuscript. Section 3 reports the subject-specific optimization simulations from the continuous infusion (red) and bolus (black) mathematical models reported in the main manuscript. Each individual simulation shown in this section (Figures S1-S29) shows the optimal model output with that subject's cytokine data plotted (either from the continuous infusion study (Berg et al., 2012) or the bolus study (Janum et al., 2016)).

Section 4 reports simulations (Figures S30-S34) of the continuous infusion model where the infusion duration and total endotoxin dose administered were increased simultaneously. The continuous infusion was given as a 4, 8, 12, 24, and 36-hour infusion, and a total endotoxin dose of 2, 4, 8, and 16 ng/kg was administered. Figure S35 displays a 2 ng/kg infusion of endotoxin over 18, 20, 32, and 36 hours. This simulation displays that the oscillatory cytokine behavior arises when the 2 ng/kg infusion is given between a 20 and 32 hour span. After this range, the oscillations are die out due to lowered endotoxin stimuli within the system. Figure S36 shows a temporal extension of Figure 9A in the main manuscript when a 2ng/kg dose of endotoxin was administered as a 4, 8, 12, and 24-hour infusion. This figure exhibits that it takes the system approximately 23 days when given shorter doses of endotoxin (4 and 8-hour infusions) and about 21 days (3 weeks) for longer doses of endotoxin (12 and 24-hour infusions) for the resting monocyte population to return to its baseline concentrations. All simulations in Section 4 were generated using the optimal mean model parameters from the 4-hour continuous infusion in the main manuscript. Section 5 shows Bayesian uncertainty results using the Delayed Rejection Adaptive Metropolis (DRAM) algorithm. We generate prediction and credible intervals for the model and also report the DRAM parameter chains, correlations, and distributions. We compare these DRAM results to the frequentist uncertainty quantification results shown in the manuscript.

S2 Mathematical background

S2.1 Scaling technique for nominal parameters

The nominal model fit to the peak magnitudes of cytokine profiles was improved by scaling the peak concentration of state $i = \{TNF, IL6, IL8, IL10\}$, denoted X_i, using the technique from Windoloski et al. (2023) where

$$X_i = \alpha \tilde{X}_i, \tag{S1}$$

for the scaling factor α and desired peak concentration $\tilde{X}_i$. We substituted equation (S1) into the ODE for state X_i (written in the main manuscript) giving

$$\frac{dX_i}{dt} = f\left(t, \theta, X_i, X_j\right) \qquad \Longrightarrow \qquad \frac{d(\alpha\tilde{X}_i)}{dt} = f\left(t, \theta, \alpha\tilde{X}_i, X_j\right)$$

for states $j \neq i$. Therefore,

$$\frac{d\tilde{X}_i}{dt} = \frac{1}{\alpha} f\left(t, \theta, \alpha\tilde{X}_i, X_j\right). \tag{S2}$$

The scaling factor $1/\alpha$ was distributed to each term on the right side of the ODE, scaling

the associated parameters in each term. State X_i was also scaled when it was upregulating another state variable, Y, as

$$H_Y^U(X_i) = H_Y^U(\alpha\tilde{X}_i) = \frac{(\alpha\tilde{X}_i)^h}{\eta_{YX_i}^h + (\alpha\tilde{X}_i)^h} \quad\Longrightarrow\quad H_Y^U(\tilde{X}_i) = \frac{\tilde{X}_i^{\,h}}{\left(\frac{\eta_{YX_i}}{\alpha}\right)^h + \tilde{X}_i^{\,h}}.$$

Thus, half-saturation values were scaled by $1/\alpha$. A similar approach was applied for down-regulation functions. Baseline cytokine parameters (w_i) were also scaled. This analysis was also applied to the nominal parameter values of individual subjects. To reduce the number of scaled parameters in the subject-specific optimizations, we only scaled cytokines with scaling factor $\alpha < 0.9$ or $\alpha > 1.1$.

S2.2 Sensitivity analysis

We conducted a local relative sensitivity analysis as described in Olufsen and Ottesen (2013) on the mean continuous infusion response using the residual vector, r, which was stated right above equation (9) in the main manuscript. The sensitivity matrix χ was given by

$$\chi = \frac{\partial r}{\partial \log(\theta)} = \frac{\partial y}{\partial \theta} \cdot \frac{\theta}{\max(y_{data})}, \tag{S3}$$

where $y = g(t, X(t), \theta)$ was the model output at time t, θ the nominal parameter set, and y_{data} the mean continuous infusion data. We approximated the (i, j) entry in the submatrix χ_k using forward differences, where

$$\chi = \begin{bmatrix} \chi_{TNF} & \chi_{IL6} & \chi_{IL8} & \chi_{IL10} \end{bmatrix}^T. \tag{S4}$$

For submatrix χ_k, elements χ_{ij} were given by

$$\chi_{ij} = \frac{g\left(t_i, X_k(t_i), \theta + he_j\right) - g\left(t_i, X_k(t_i), \theta\right)}{h} \frac{\theta}{\max\left(y_{data}^k\right)}, \tag{S5}$$

where $\phi = 10^{-8}$ was the solver tolerance, $h = \sqrt{\phi}$ the step size (Pope et al., 2009), and e_j the basis vector in the jth direction. As stated in the main manuscript, we ranked relative sensitivities by computing the two-norm of each column of χ, obtaining a single sensitivity per parameter. We repeated the sensitivity analysis by simulating 100 runs sampling parameters from a uniform distribution varying $\pm 30\%$ around the parameter's nominal value to study effects due to perturbations in parameter values.

S2.3 Subset selection

We used two practical identifiability techniques, the structured correlation method (SCM) and the singular value decomposition (SVD) QR method (Miao et al., 2011; Olufsen and Ottesen, 2013). The SCM used the Fisher-information matrix $F = \chi^T\chi$. We checked the condition number to ensure that F had an inverse and calculated $G = F^{-1}$. The matrix G was used to determine the pairwise parameter covariance C_{ij} by

$$C_{ij} = \frac{G_{ij}}{\sqrt{G_{ii}\,G_{jj}}}, \tag{S6}$$

where (i, j) refers to θ_i and θ_j. Parameter pairs for which $|C_{ij}| > 0.9$ were considered correlated. The parameter set with the largest correlation was selected. The parameter within that set with the smallest relative sensitivity was removed from the parameter set, and the process was repeated until there were no correlated parameters.

The SVD-QR method used singular value decomposition (SVD) to determine identifiable parameters. This method decomposed the sensitivity matrix $\chi = U\Sigma V^T$ where U and V contained the left and right singular vectors of χ, and Σ contained the singular values σ of χ. The largest k singular values of χ were determined by $\sigma(k) \geq 10\sqrt{\phi}$ where ϕ was the ODE solver tolerance. The first k columns of the right singular vectors, V_k, were extracted from V and used to find a permutation matrix P such that $V_k^T P = QR$, where Q was an orthogonal matrix and R was an upper triangular matrix. The permutation matrix P was then used to reorder the parameter vector θ as

$$\hat{\theta} = P^T\theta. \tag{S7}$$

The first k parameters of $\hat{\theta}$ were considered identifiable. The identifiable parameter sets were then subject to additional analysis using the coefficient of variation, as described in the main manuscript.

S2.4 Goodness of fits

We calculated three goodness of fit measurements for each parameter subset in the main manuscript by computing the coefficient of determination (R^2) (Dodge, 2008), the corrected Akaike information criterion (AICc) (Burnham and Anderson, 2002), and the Bayesian information criterion (BIC) (Schwarz, 1978). The coefficient of determination for cytokines $k \in$

$\{TNF, IL6, IL8, IL10\}$ was given by

$$R_k^2 = 1 - \frac{SSE_k}{SST_k}.$$

SSE_k was the sum of squared error of cytokine k and was given by

$$SSE_k = \sum_{i=1}^{N} \left(y_{data}^k(t_i) - \tilde{y}_i^k\right)^2$$

where $y_{data}^k(t_i)$ was the data for cytokine k at time t_i for $1 \leq i \leq N$ and $\tilde{y}_i^k = g(t_i, X_k(t_i), \tilde{\theta})$ was the optimized model output for cytokine k at time t_i. SST was the sum of squared error given by

$$SST_k = \sum_{i=1}^{N} \left(y_{data}^k(t_i) - \bar{y}_{data}^k\right)^2$$

where $\bar{y}_{data}^k$ was the mean of the observed data from cytokine k. Larger R^2 values indicated a better explanation of the data by the model. The AICc and BIC accounted for the number of parameters being estimated. Smaller values of the AICc and BIC indicated a balance between model fit and the number of estimated parameters. AICc and BIC were calculated as

$$AICc = 2\ln(J) + 2\rho + \frac{2\rho(\rho+1)}{M-\rho-1}$$

and

$$BIC = 2\ln(J) + \rho\ln(M)$$

where J was the least squares cost as defined above equation (9) in the main manuscript, ρ the number of estimated parameters, and M the total number of data points used in model calibration for each parameter subset.

S2.5 Confidence and prediction intervals

We constructed parameter and model confidence and prediction intervals using the frequentist approach detailed in Seber and Wild (2003); Banks et al. (2009); Smith (2013). Parameter confidence intervals for optimized parameter $\tilde{\theta}_i$ were computed as

$$\tilde{\theta}_i \pm t_{N-q}^{\alpha/2}\sqrt{\Sigma_{ii}}, \quad \text{(S8)}$$

where N was the total number of data points, q was the number of parameters that were estimated, $t_{N-q}^{\alpha/2}$ was the t-value from the student's t-distribution for confidence level $1-\alpha$ with $N-q$ degrees of freedom, and the variance estimator matrix Σ was given by

$$\Sigma = (\chi^T(\tilde{\theta})V^{-1}\chi(\tilde{\theta}))^{-1}. \tag{S9}$$

We defined χ similarly to equations (S4) and (S5) where

$$\chi(\tilde{\theta}) = \begin{bmatrix} \chi_{TNF}(\tilde{\theta}) & \chi_{IL6}(\tilde{\theta}) & \chi_{IL8}(\tilde{\theta}) & \chi_{IL10}(\tilde{\theta}) \end{bmatrix}^T \tag{S10}$$

and the (i,j) element of submatrix $\chi_k(\tilde{\theta})$ with $k \in \{TNF, IL6, IL8, IL10\}$ was approximated using forward differences given by

$$\chi_{ij}(\tilde{\theta}) = \frac{g\left(t_i, X_k(t_i), \tilde{\theta} + he_j\right) - g\left(t_i, X_k(t_i), \tilde{\theta}\right)}{h}. \tag{S11}$$

Here, $\tilde{y}_i^k = g(t_i, X_k(t_i), \tilde{\theta})$ was the optimal model output with optimal parameter vector $\tilde{\theta}$ for cytokine state k at time t_i for $1 \leq i \leq N_k$ where N_k was the number data points for cytokine k, and h and e_j were defined as in equation (S5). The diagonal variance matrix V was given by

$$V = \text{diag}\left(\sigma_{TNF}, \sigma_{IL6}, \sigma_{IL8}, \sigma_{IL10}\right), \tag{S12}$$

where σ_k was a diagonal matrix of size $N_k \times N_k$ with entries

$$\frac{1}{N_k - q}\left(r_k^T r_k\right), \quad r_k = \left[\tilde{y}_1^k \ldots \tilde{y}_{N_k}^k\right] - y_{data}^k \tag{S13}$$

with y_{data}^k defined in equation (9) in the main manuscript. The asymptotic prediction interval for cytokine k at time t_i was given by

$$PI_i^k = \tilde{y}_i^k \pm t_{N_k-q_k}^{\alpha/2} s_k \sqrt{1 + \mathbf{G}_{ik}^T \left(\chi_k^T(\tilde{\theta})\chi_k(\tilde{\theta})\right)^{-1} \mathbf{G}_{ik}} \tag{S14}$$

and the confidence interval by

$$CI_i^k = \tilde{y}_i^k \pm t_{N_k-q_k}^{\alpha/2} s_k \sqrt{\mathbf{G}_{ik}^T \left(\chi_k^T(\tilde{\theta})\chi_k(\tilde{\theta})\right)^{-1} \mathbf{G}_{ik}}. \tag{S15}$$

We defined $\tilde{y}_i^k$ and N_k as in equation (S11), q_k was the number of estimated parameters that impacted cytokine state k, and $t_{N_k-q_k}^{\alpha/2}$ was the t-value for confidence level $1-\alpha$ with $N_k - q_k$

degrees of freedom. $\chi_k(\tilde{\theta})$ was given by (S10) and (S11), but as stated in the main manuscript, columns in $\chi_{TNF}(\tilde{\theta})$, $\chi_{IL6}(\tilde{\theta})$, and $\chi_{IL10}(\tilde{\theta})$ corresponding to IL-8 parameters were eliminated since they did not impact those state variables (see Figure 4 in the main manuscript). Entries of these columns were approximately zero and made $F_k = \chi_k^T(\tilde{\theta})\chi_k(\tilde{\theta})$ singular unless removed. The matrix $\mathbf{G}_{ik}^T$ was defined as

$$\mathbf{G}_{ik}^T = \left(\frac{\partial \tilde{y}_i^k}{\partial \theta_1} \cdots \frac{\partial \tilde{y}_i^k}{\partial \theta_{q_k}} \right), \tag{S16}$$

which was ith row of the submatrix $\chi_k(\tilde{\theta})$, and the variance estimator s_k^2 was given by

$$s_k^2 = \frac{1}{N_k - q_k} r_k^T r_k, \tag{S17}$$

with r_k in equation (S13).

S3 Subject-specific optimizations

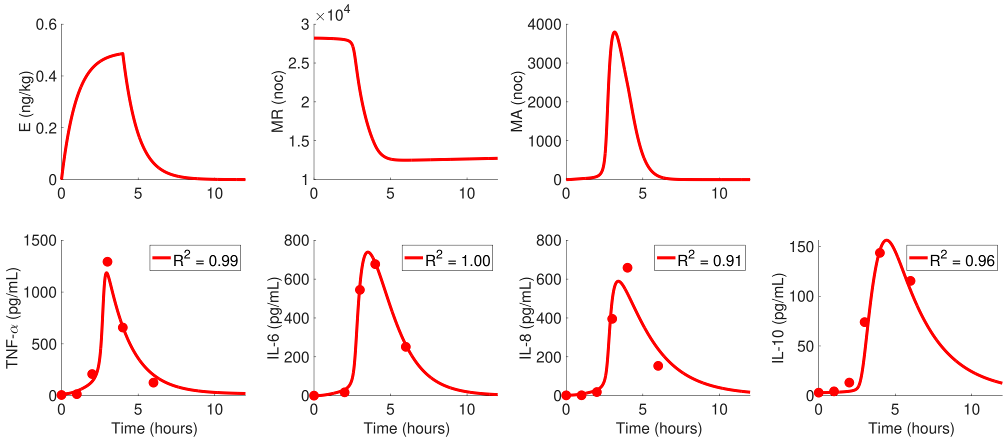

Figure S1: Model fit for subject 1 from the continuous infusion subject-specific optimization.

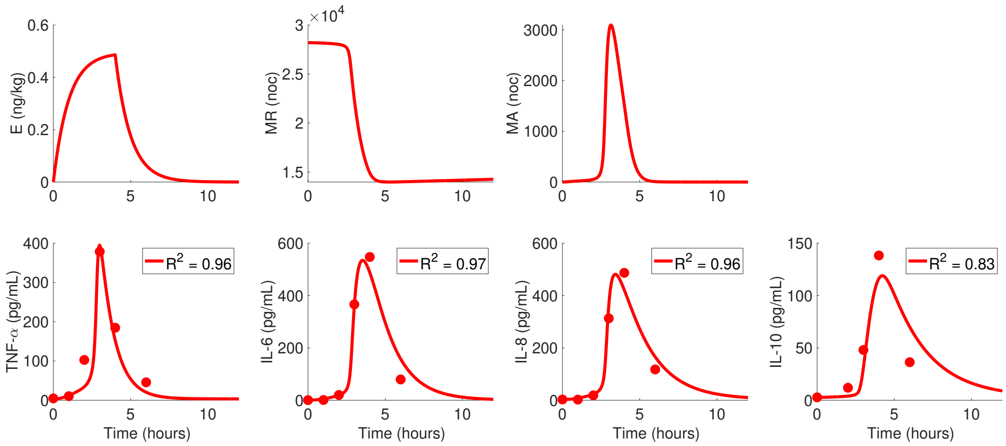

Figure S2: Model fit for subject 2 from the continuous infusion subject-specific optimization.

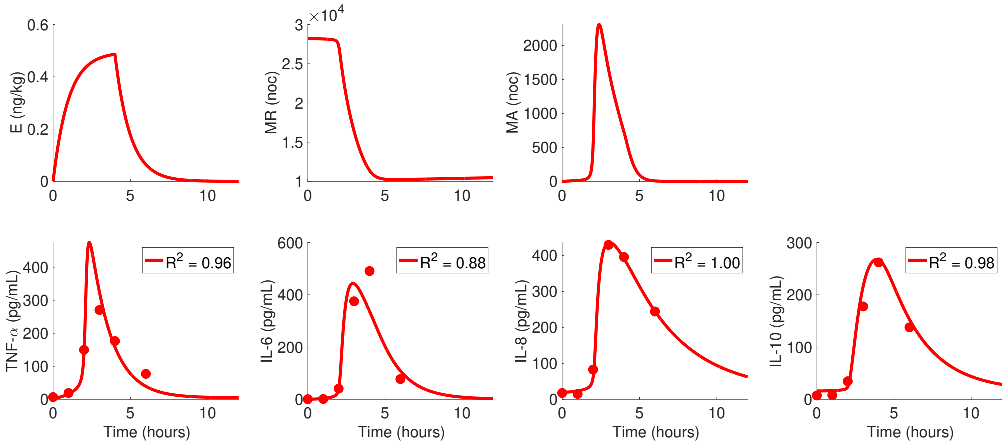

Figure S3: Model fit for subject 3 from the continuous infusion subject-specific optimization.

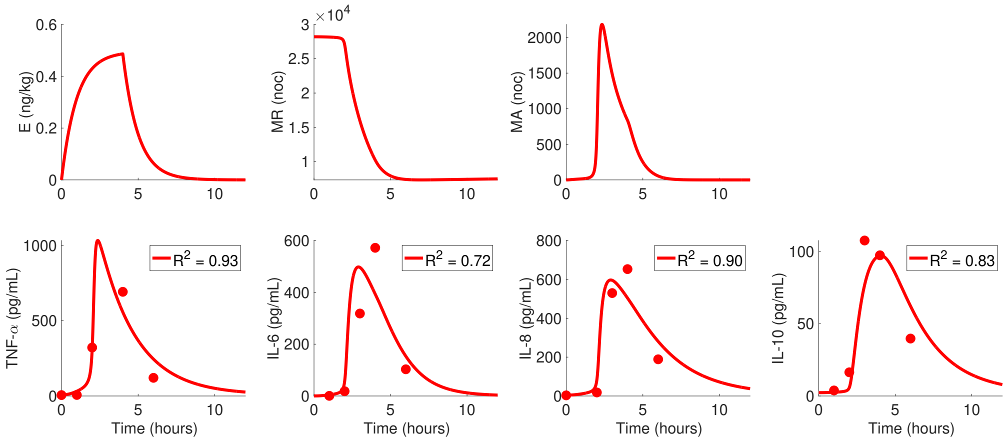

Figure S4: Model fit for subject 4 from the continuous infusion subject-specific optimization.

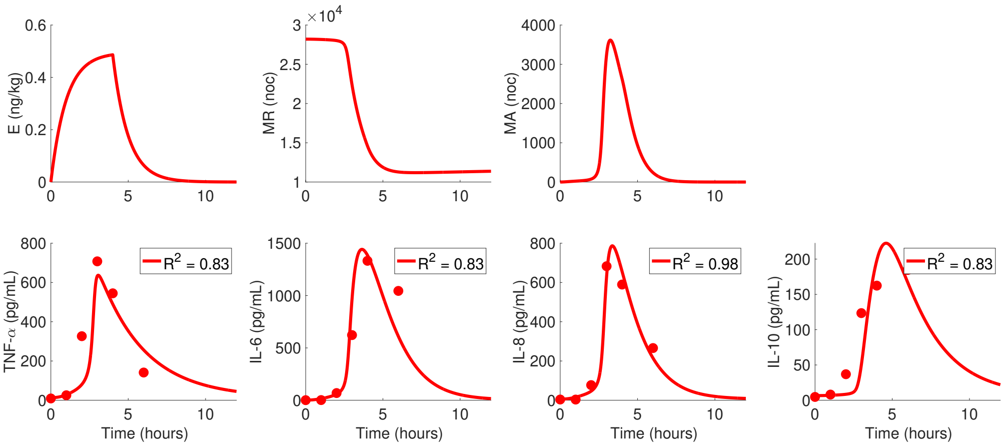

Figure S5: Model fit for subject 5 from the continuous infusion subject-specific optimization.

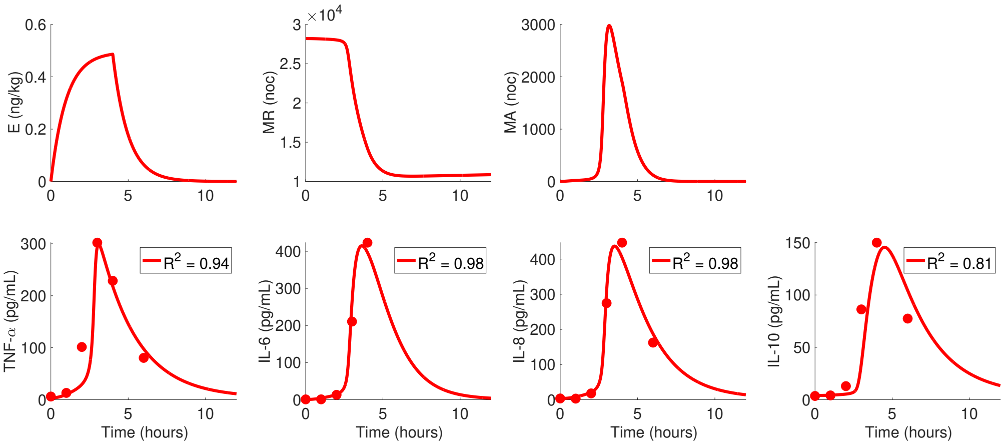

Figure S6: Model fit for subject 6 from the continuous infusion subject-specific optimization.

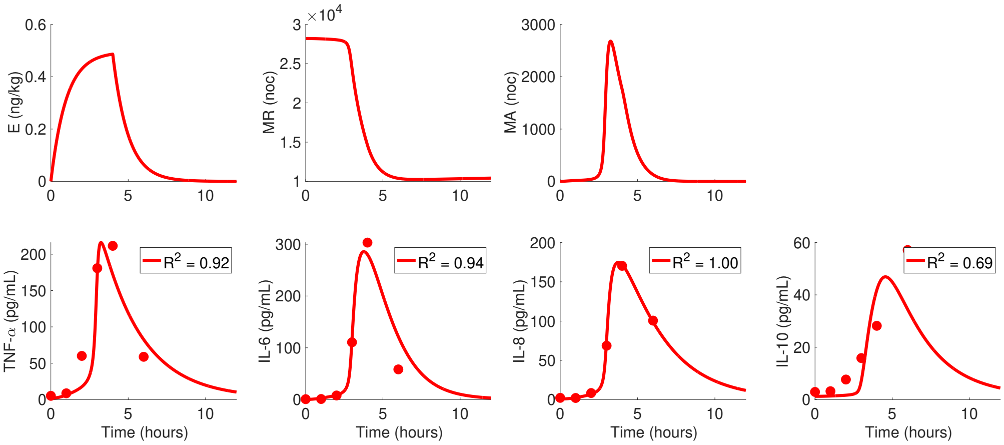

Figure S7: Model fit for subject 7 from the continuous infusion subject-specific optimization.

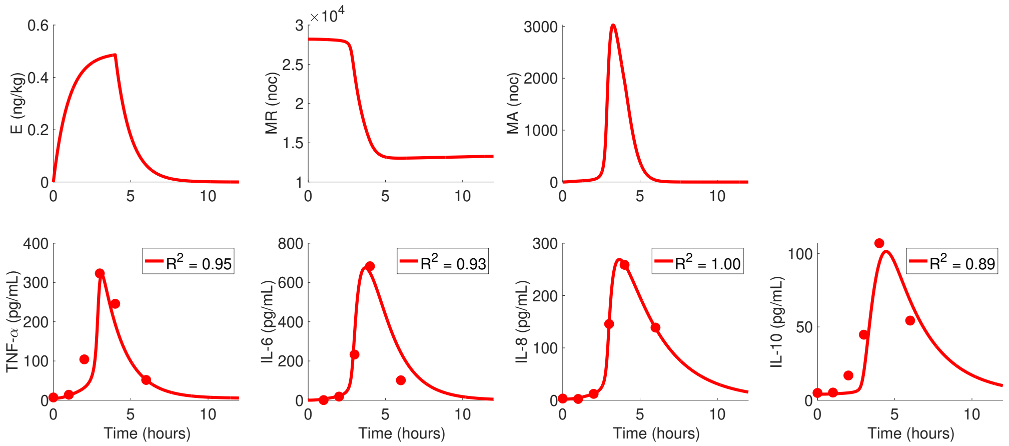

Figure S8: Model fit for subject 8 from the continuous infusion subject-specific optimization.

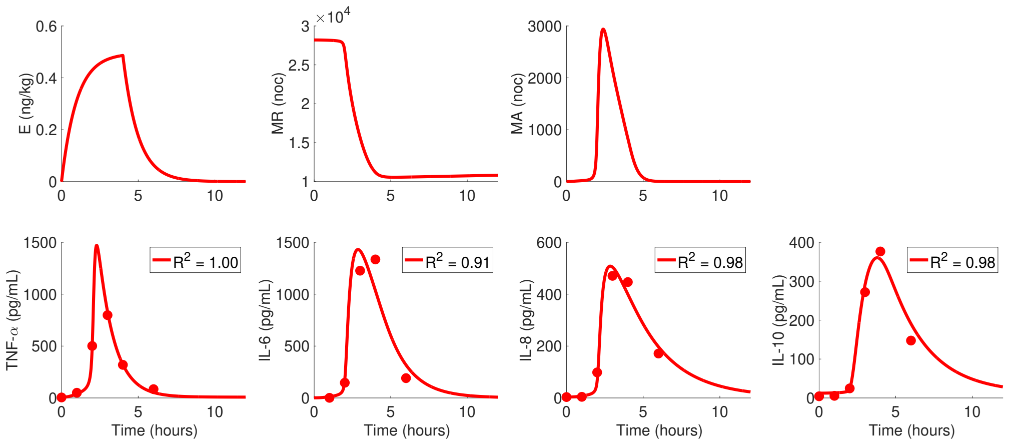

Figure S9: Model fit for subject 9 from the continuous infusion subject-specific optimization.

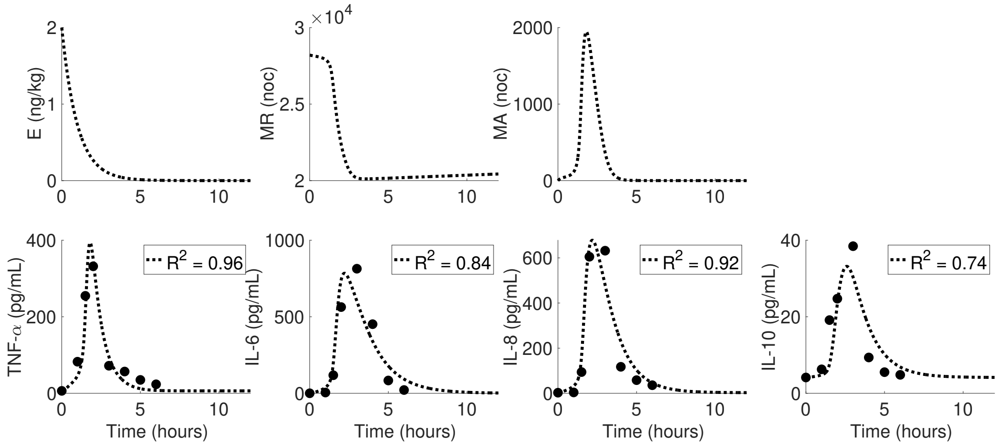

Figure S10: Model fit for subject 1 from the bolus subject-specific optimization.

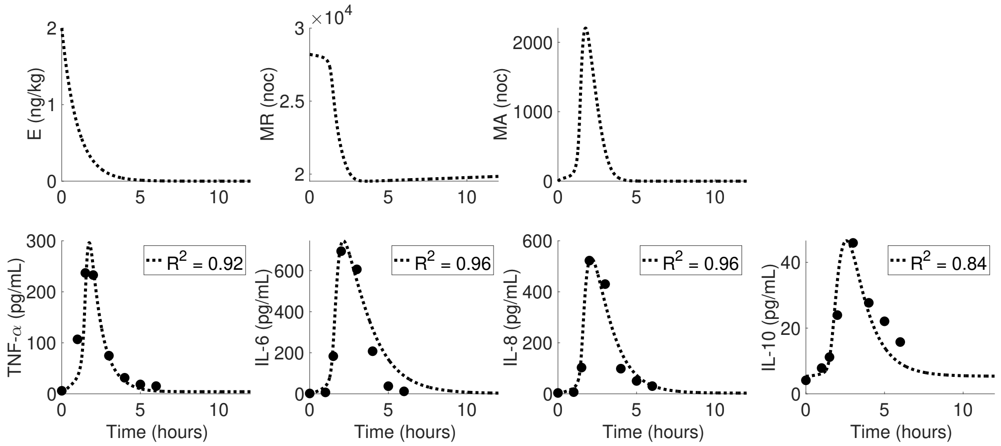

Figure S11: Model fit for subject 2 from the bolus subject-specific optimization.

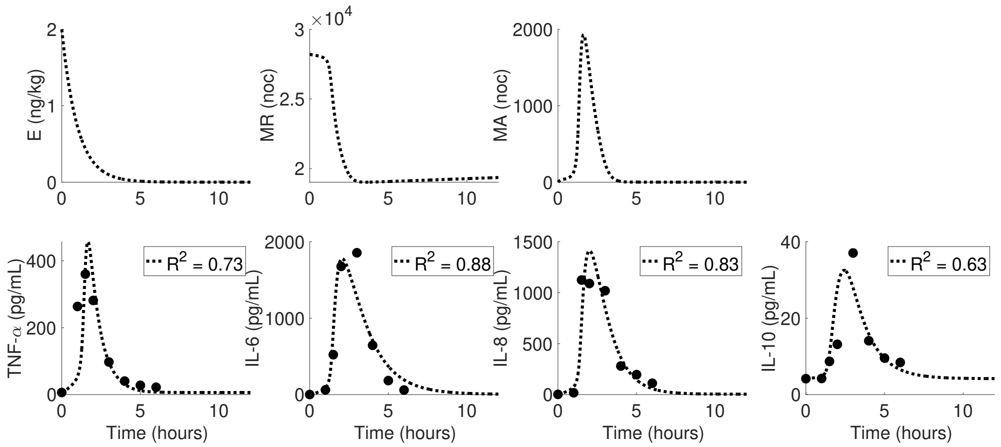

Figure S12: Model fit for subject 3 from the bolus subject-specific optimization.

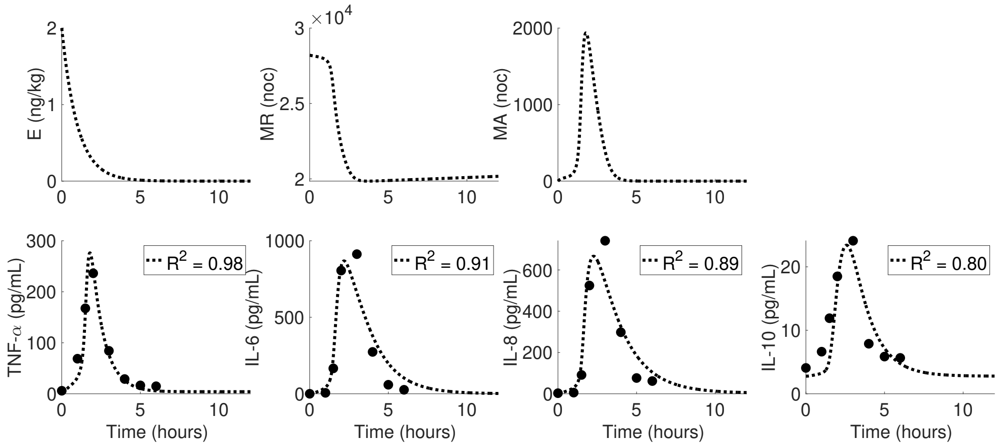

Figure S13: Model fit for subject 4 from the bolus subject-specific optimization.

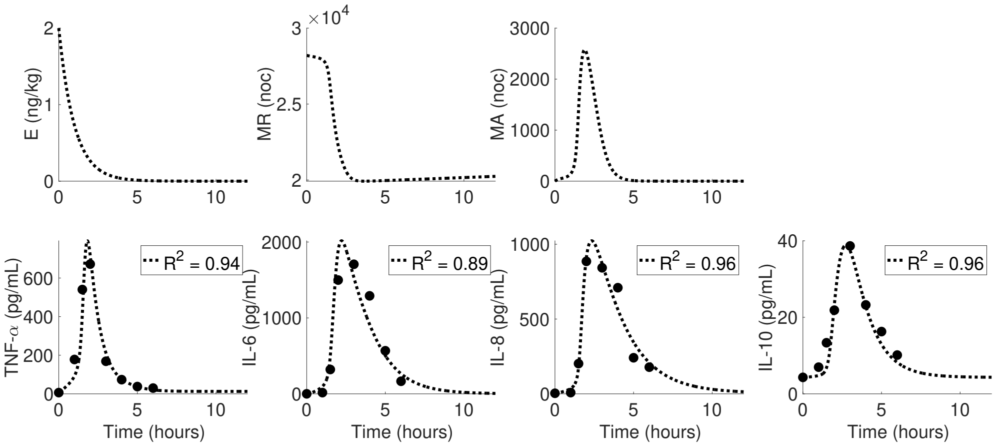

Figure S14: Model fit for subject 5 from the bolus subject-specific optimization.

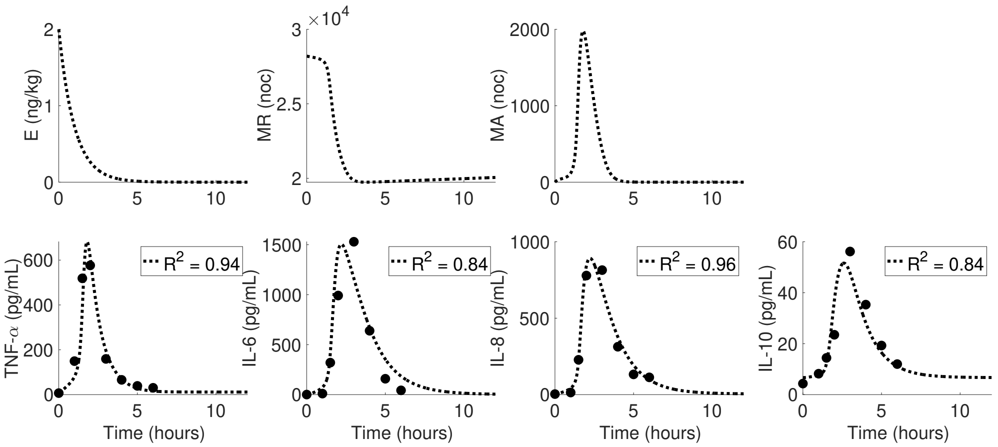

Figure S15: Model fit for subject 6 from the bolus subject-specific optimization.

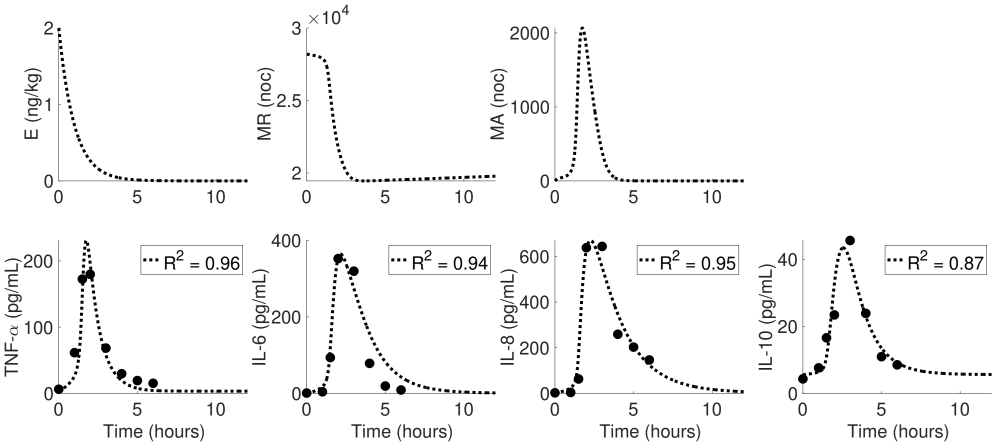

Figure S16: Model fit for subject 7 from the bolus subject-specific optimization.

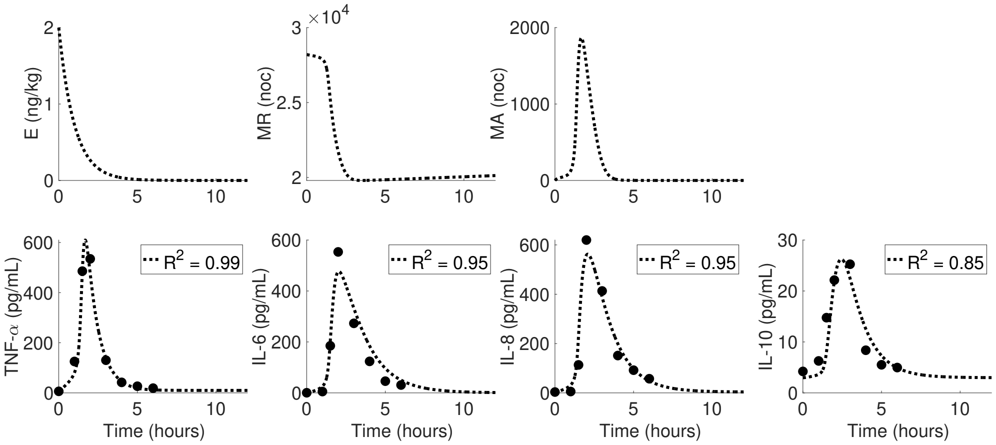

Figure S17: Model fit for subject 8 from the bolus subject-specific optimization.

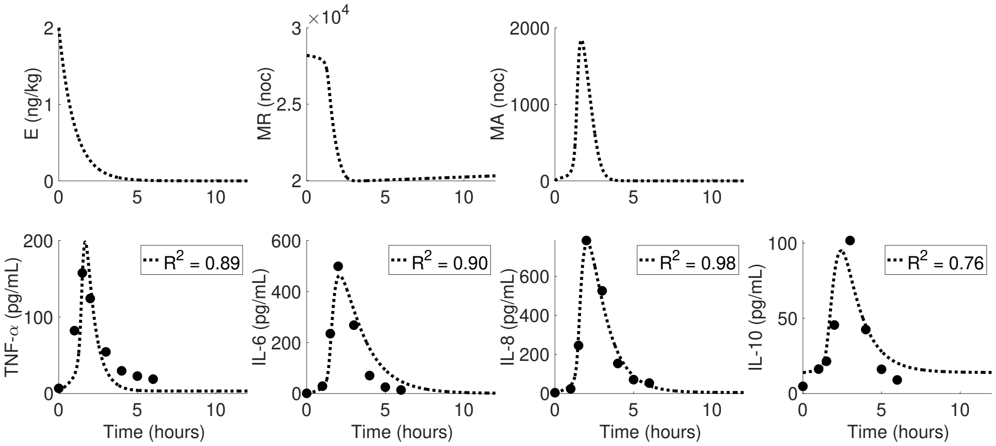

Figure S18: Model fit for subject 9 from the bolus subject-specific optimization.

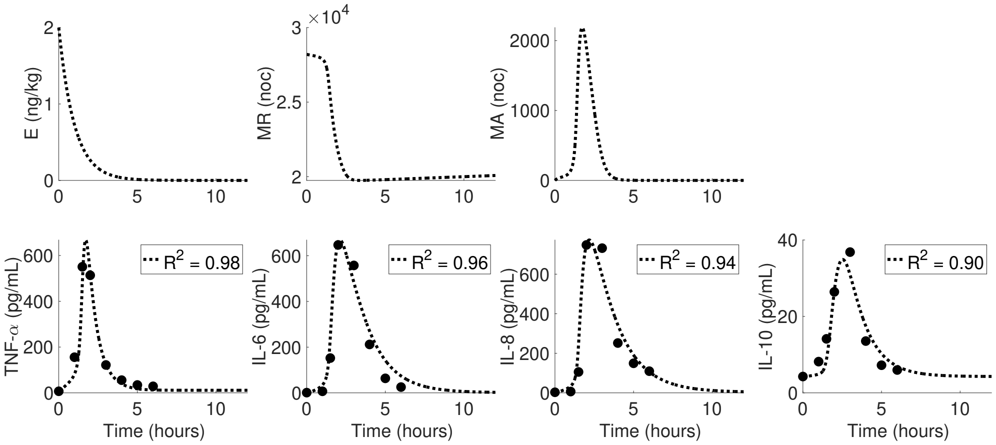

Figure S19: Model fit for subject 10 from the bolus subject-specific optimization.

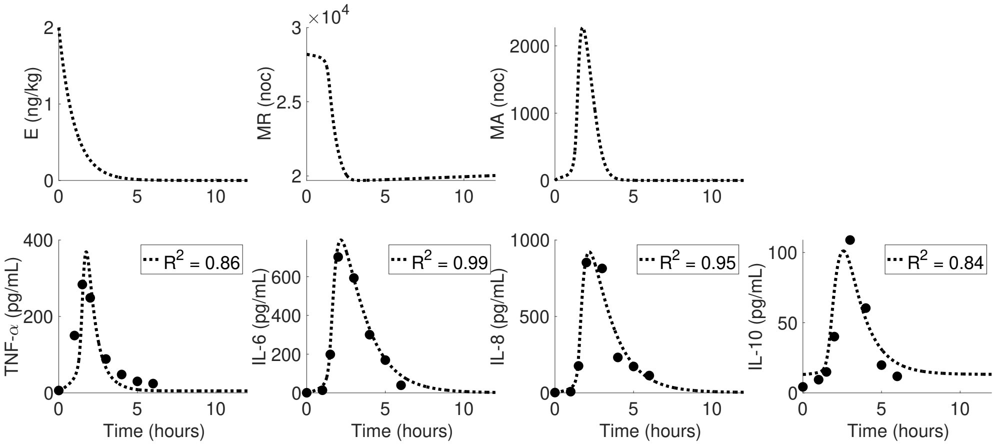

Figure S20: Model fit for subject 11 from the bolus subject-specific optimization.

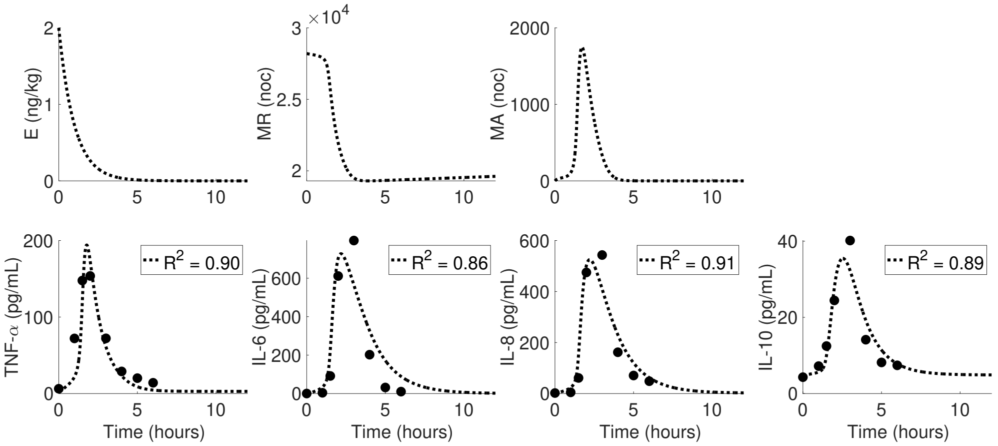

Figure S21: Model fit for subject 12 from the bolus subject-specific optimization.

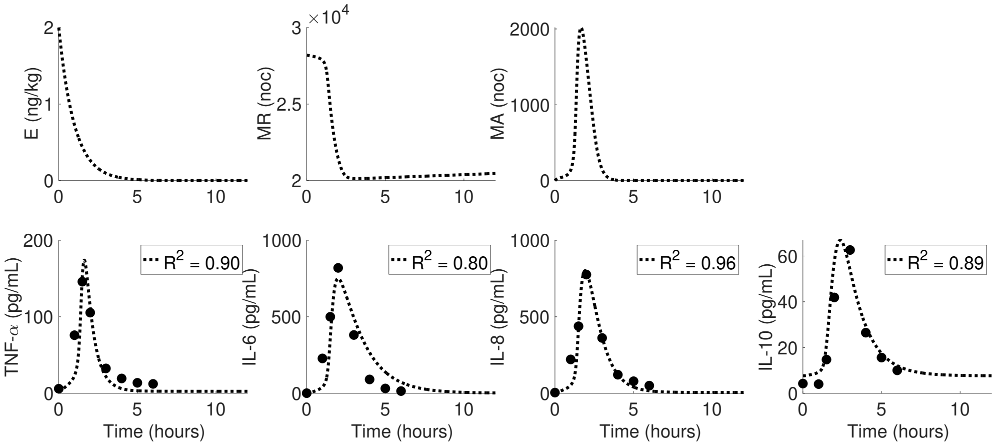

Figure S22: Model fit for subject 13 from the bolus subject-specific optimization.

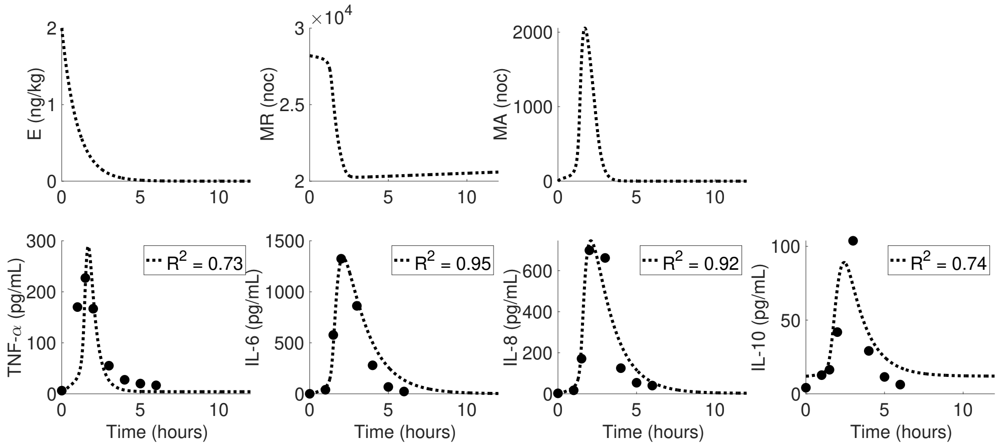

Figure S23: Model fit for subject 14 from the bolus subject-specific optimization.

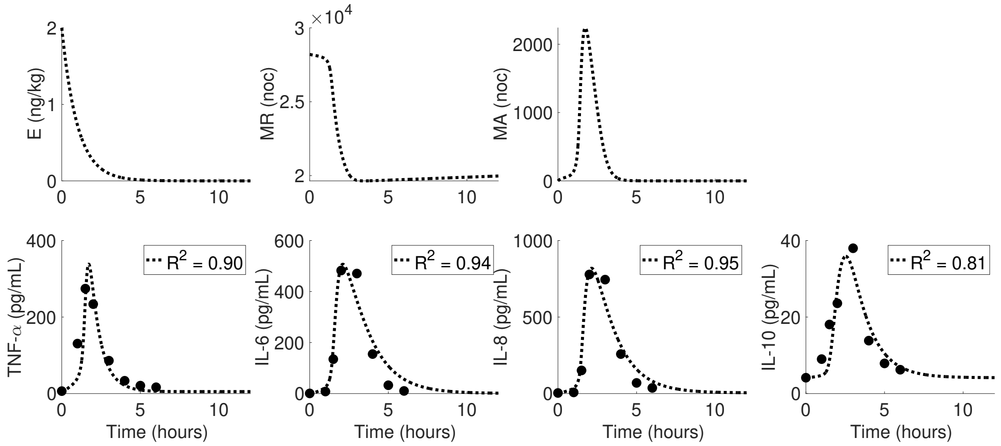

Figure S24: Model fit for subject 15 from the bolus subject-specific optimization.

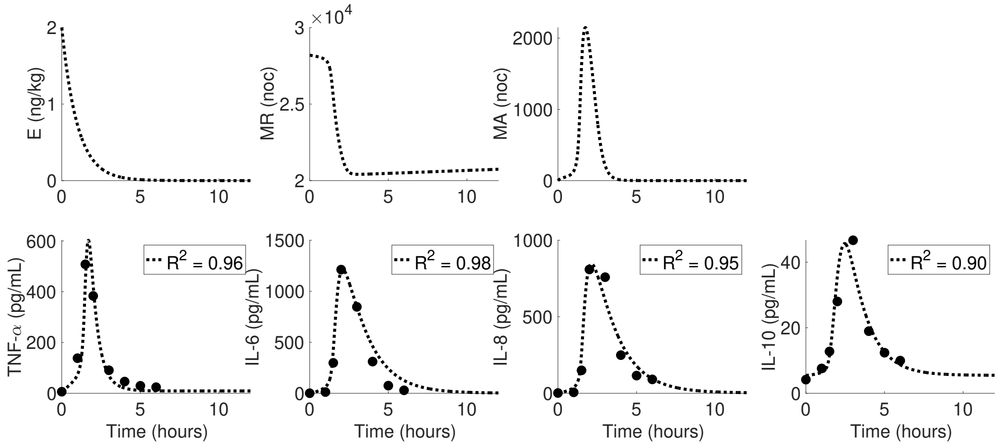

Figure S25: Model fit for subject 16 from the bolus subject-specific optimization.

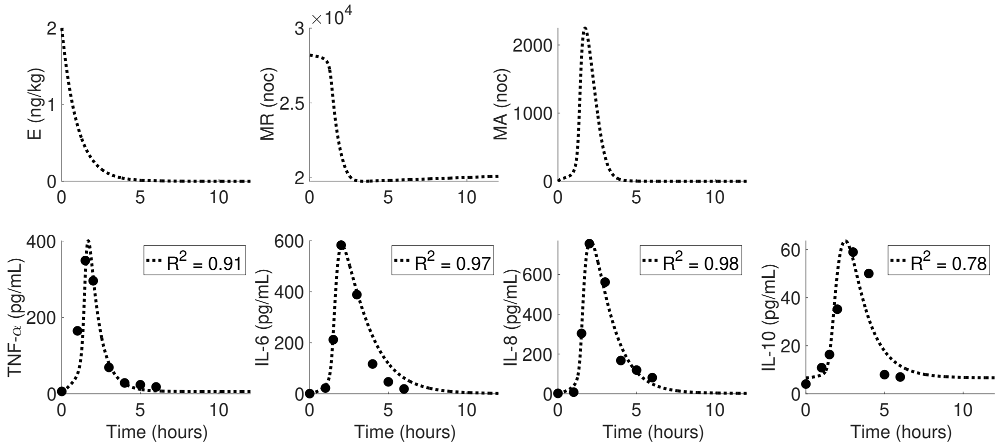

Figure S26: Model fit for subject 17 from the bolus subject-specific optimization.

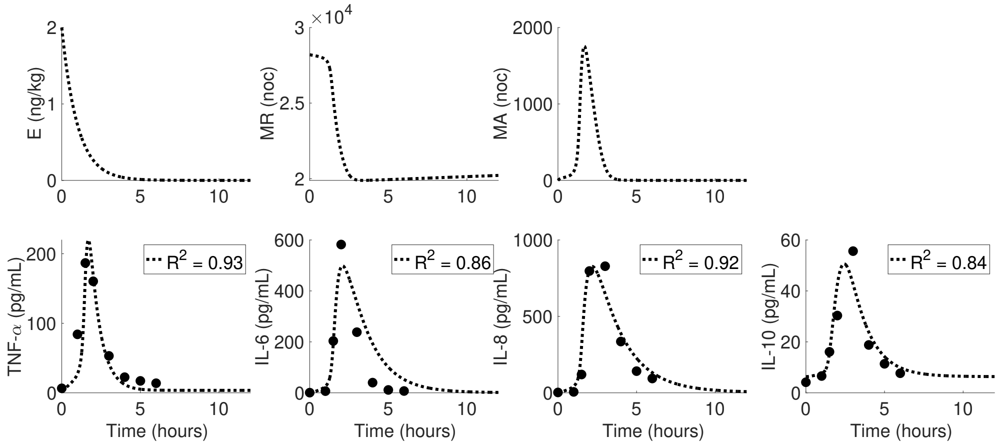

Figure S27: Model fit for subject 18 from the bolus subject-specific optimization.

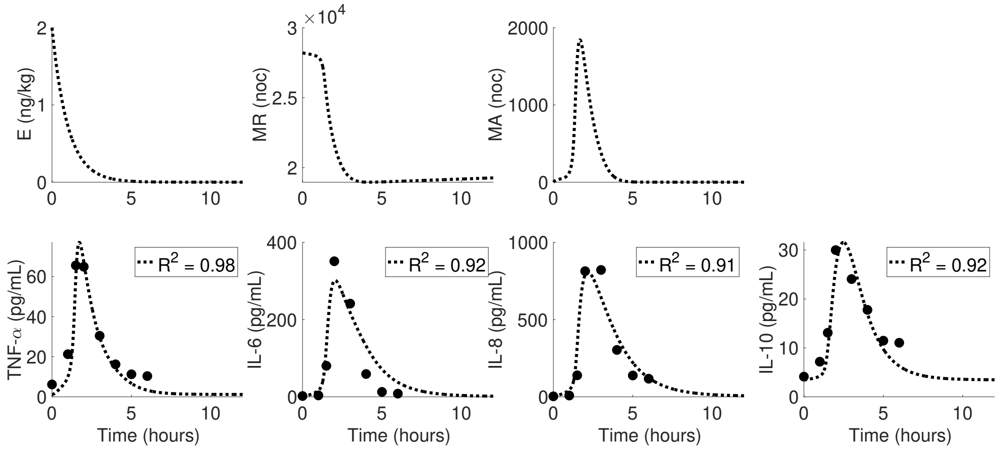

Figure S28: Model fit for subject 19 from the bolus subject-specific optimization.

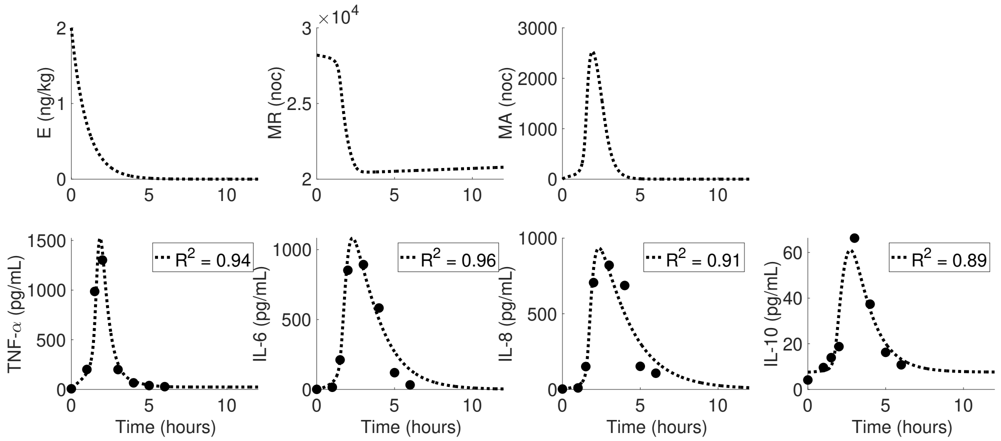

Figure S29: Model fit for subject 20 from the bolus subject-specific optimization.

S4 Endotoxin perturbations

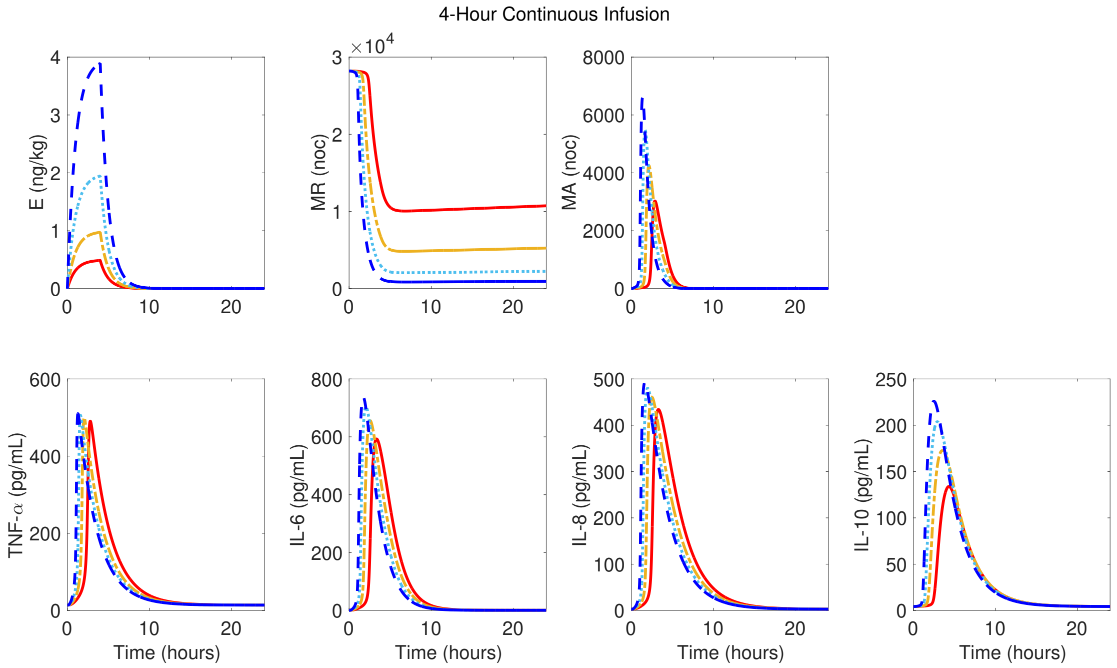

Figure S30: 4-hour continuous infusion of 2 ng/kg (red solid lines), 4 ng/kg (yellow dash-dotted lines), 8 ng/kg (light blue dotted lines), and 16 ng/kg (dark blue dashed lines) of endotoxin using the optimal mean model parameters.

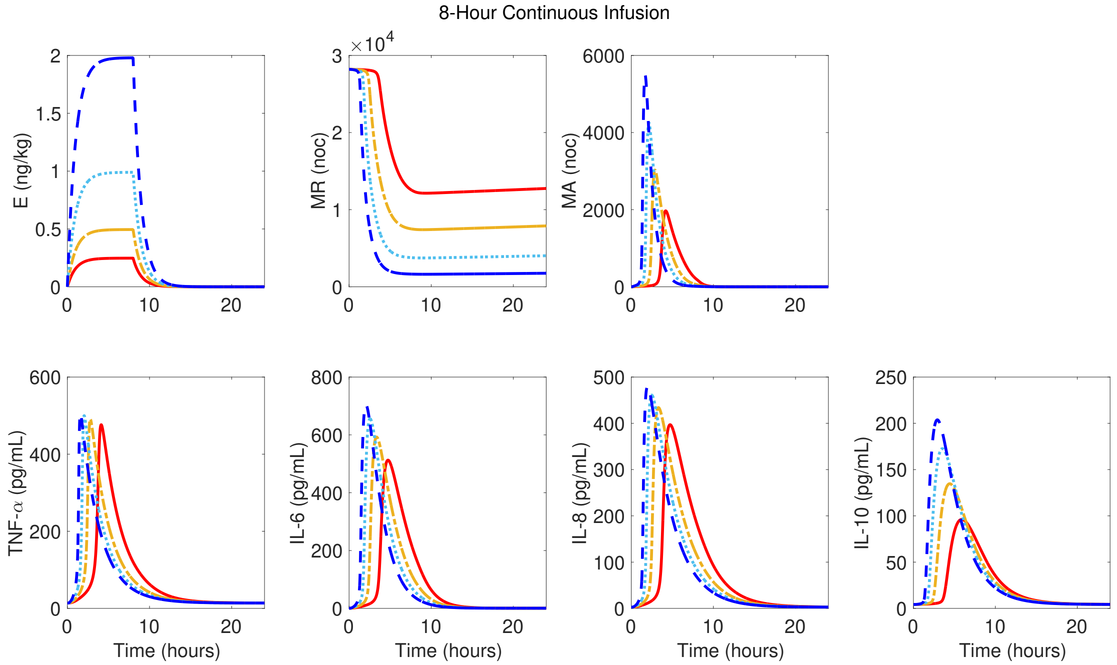

Figure S31: 8-hour continuous infusion of 2 ng/kg (red solid lines), 4 ng/kg (yellow dash-dotted lines), 8 ng/kg (light blue dotted lines), and 16 ng/kg (dark blue dashed lines) of endotoxin using the optimal mean model parameters.

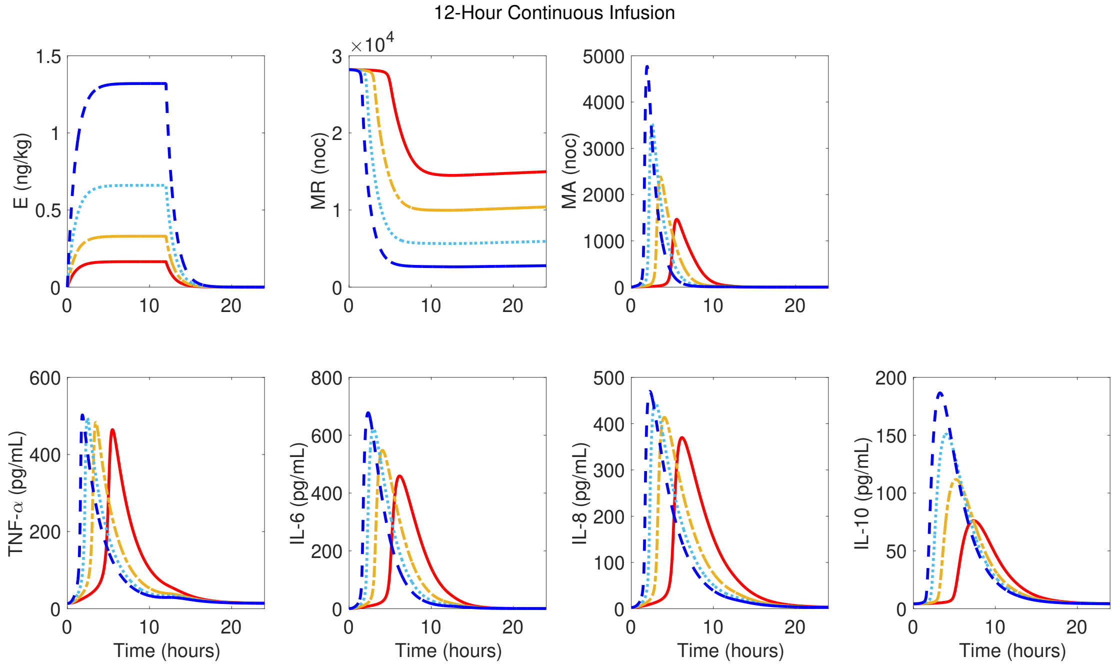

Figure S32: 12-hour continuous infusion of 2 ng/kg (red solid lines), 4 ng/kg (yellow dash-dotted lines), 8 ng/kg (light blue dotted lines), and 16 ng/kg (dark blue dashed lines) of endotoxin using the optimal mean model parameters.

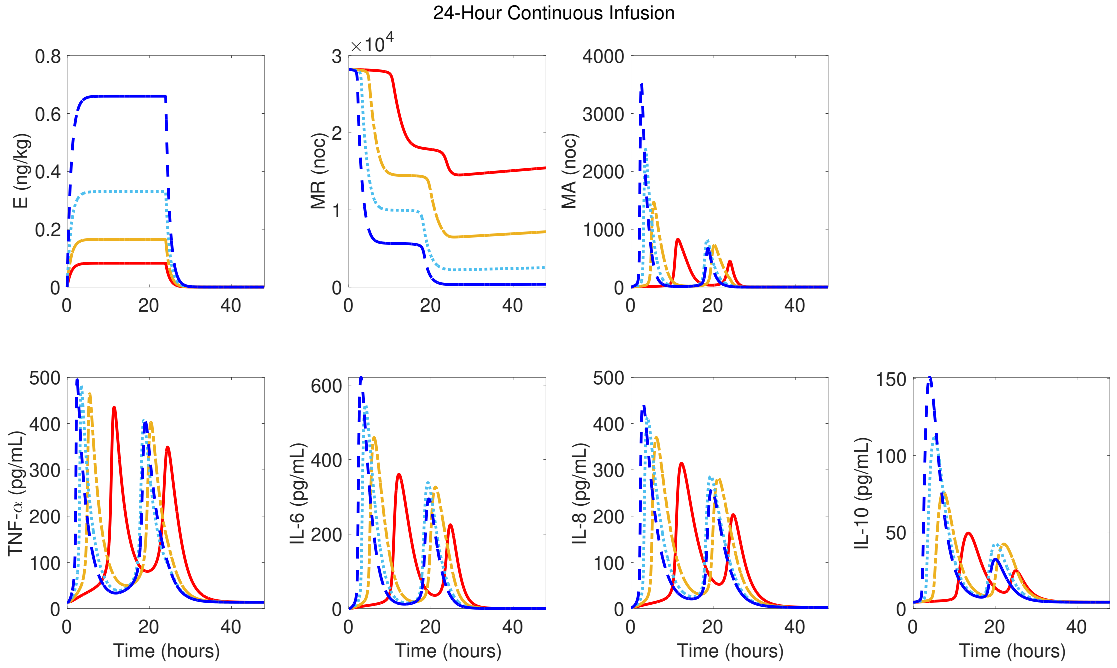

Figure S33: 24-hour continuous infusion of 2 ng/kg (red solid lines), 4 ng/kg (yellow dash-dotted lines), 8 ng/kg (light blue dotted lines), and 16 ng/kg (dark blue dashed lines) of endotoxin using the optimal mean model parameters.

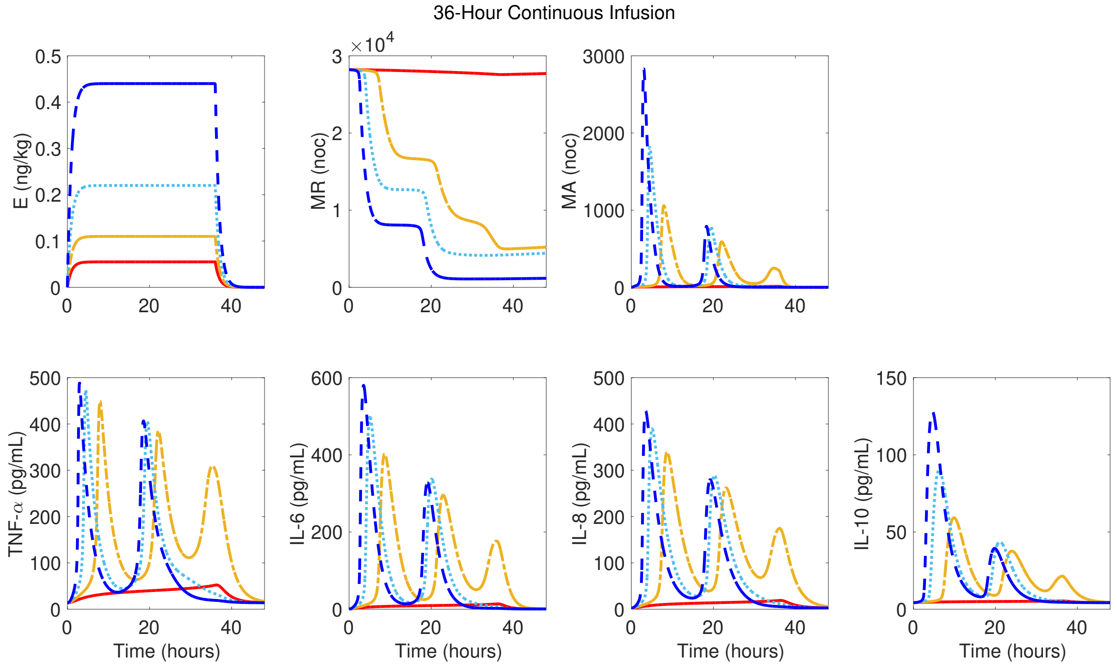

Figure S34: 36-hour continuous infusion of 2 ng/kg (red solid lines), 4 ng/kg (yellow dash-dotted lines), 8 ng/kg (light blue dotted lines), and 16 ng/kg (dark blue dashed lines) of endotoxin using the optimal mean model parameters.

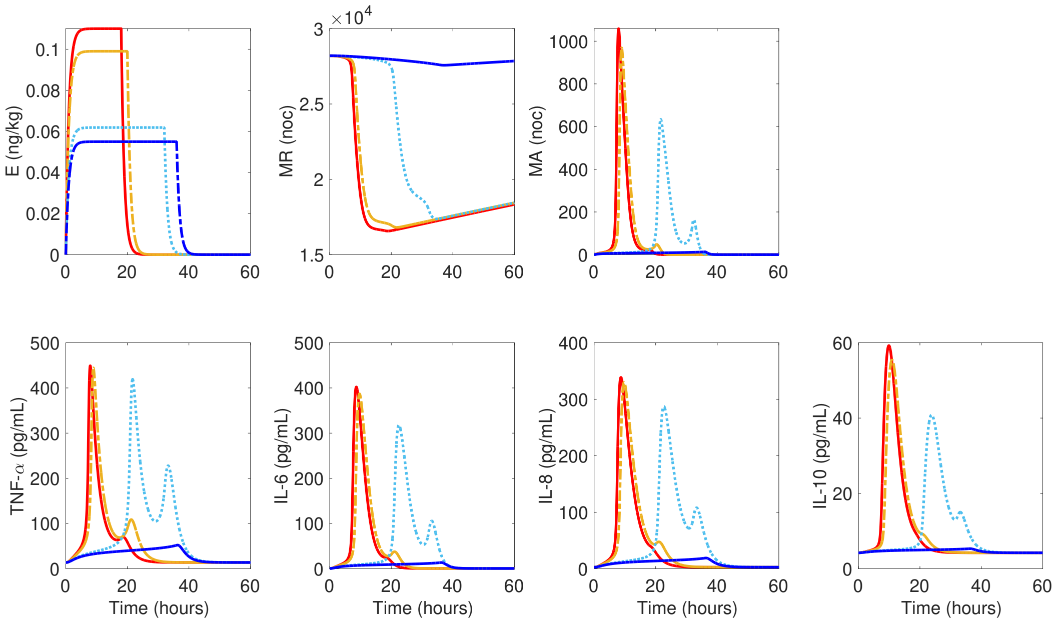

Figure S35: 2 ng/kg continuous infusion for 18 (red solid lines), 20 (yellow dash-dotted lines), 32 (light blue dotted lines), and 36 hours (dark blue dashed lines) using the optimal mean model parameters.

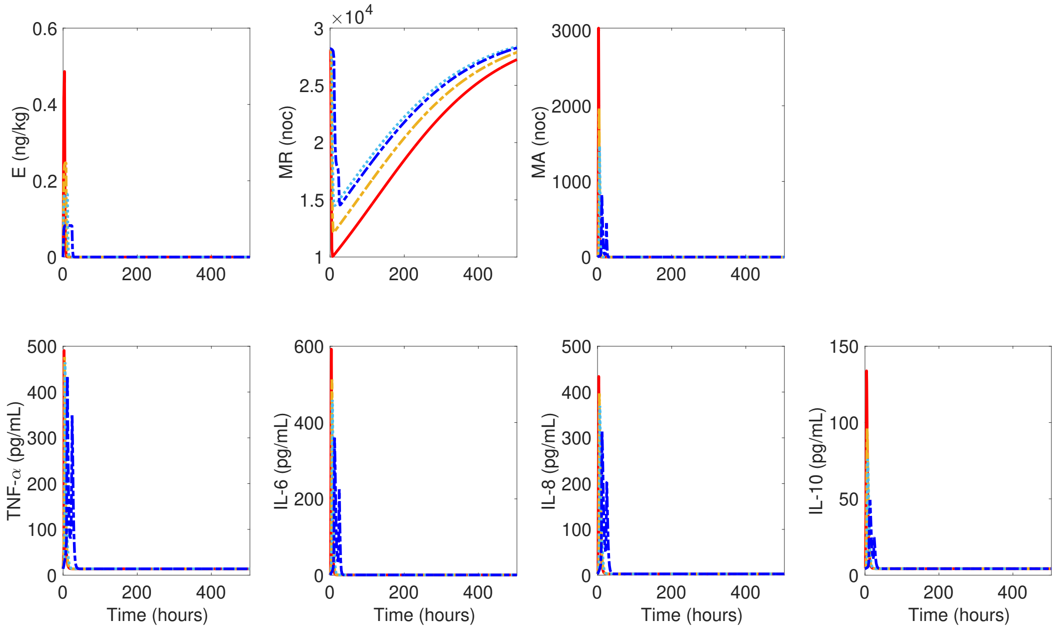

Figure S36: 2 ng/kg continuous infusion for four (red solid lines), eight (yellow dash-dotted lines), 16 (light blue dotted lines), and 24 hours (dark blue dashed lines) using the optimal mean model parameters. Monocytes return to baseline about 3 weeks after the infusion start for large doses of endotoxin.

S5 Delayed Rejection Adaptive Metropolis (DRAM)

We compare the asymptotic (frequentist) measures of uncertainty in Figure 7 of the main manuscript (prediction and confidence intervals) with Bayesian measures of uncertainty (prediction and credible intervals). Frequentist methods establish uncertainty by fixing parameters and assigning probabilities to data and Bayesian methods determine uncertainty through a single data set by assigning probability distributions to parameters (Fornacon-Wood et al., 2022). We generate Bayesian inference results using a sampling method called Delayed Rejection Adaptive Metropolis (DRAM), which combines two Metropolis-Hastings Markov chain Monte Carlo (MCMC) algorithms (Delayed Rejection and Adaptive Metropolis). Additional details of DRAM can be found in Haario et al. (2006); Smith (2013); Lye et al. (2021).

DRAM was run for 200,000 samples with a 40,000 burn-in period. Prediction and credible intervals for the mean bolus and continuous infusion models are shown in Figure S37. These results are similar to the frequentist prediction and confidence intervals generated in Figure 7 of the original manuscript. The credible interval is the Bayesian form of a frequentist confidence interval. It is defined as the range in which 95$\%$ of the posterior distribution lies, or the range of values that a parameter lies within with probability 0.95 (Chihara and Hesterberg, 2011). The prediction intervals for both uncertainty methods produce similar sized intervals for both models, with the frequentist prediction intervals being slightly wider than those produced by DRAM. The credible intervals for both the bolus and continuous infusion models are wider than the confidence intervals from the frequentist approach, specifically between hours zero and eight. However, the tails of both the Bayesian credible and frequentist confidence intervals are of similar width.

The parameter chains, correlations, and distributions for the DRAM simulations are shown in Figures S38-S43. The parameter chains show the convergence of DRAM following the burn-in period (marked by the vertical black line), where the parameter chain for the bolus model (Figure S38) exhibits wider bounds for all optimized parameters except for k_{TNFM} compared to the continuous infusion model chain (Figure S39), which is about the same width as k_{TNFM} in the continuous infusion. The difference in parameter chain characteristics is hypothesized to be due to the sensitive and identifiable parameter subset being derived from the continuous infusion model instead of the bolus model, leading to a larger variation in bolus model results. The DRAM parameter correlations are shown for the bolus model in Figure S40 and the continuous model in Figure S41. The densities for the continuous infusion look well distributed with possible linear correlations between parameters k_{MA} and k_{TNFM}, but this interestingly did not impact the parameter distributions shown in Figure S43. The DRAM parameter correlations for the bolus model (Figure S40) have wider, more scattered distributions and a similar linear

trend between parameters k_{MA} and k_{TNFM}. Additionally, the parameter correlations show that some parameters may being hitting their preset bounds, which is observed in the parameter chains for the bolus model (Figure S38).

Finally, the optimized parameter distributions are shown in Figures S42 and S43. All parameter distributions for both the bolus and continuous infusion models are approximately normally distributed, with the parameter distributions from the bolus model exhibiting slightly skewed behavior with longer tails. The parameter distributions from the continuous infusion model (Figure S43) show the optimal parameter value (denoted by the black line) close to the peak of the distribution curve, while the bolus model parameter distributions (Figure S42) have slightly off-peak optimal values for parameters k_{MA} and k_8 and further off-peak optimal value for parameter k_{TNF}. As previously discussed, we attribute the off-peak optimal values of several of the bolus parameters due to the optimized subset being generated from the continuous infusion. Furthermore, we suspect that the off-center optimal value for k_{TNF} occurred because that parameter is the least sensitive off all optimized parameters (shown in Figure 5 in the original manuscript). Therefore, it can vary more than the other parameters with less of an impact on the model output.

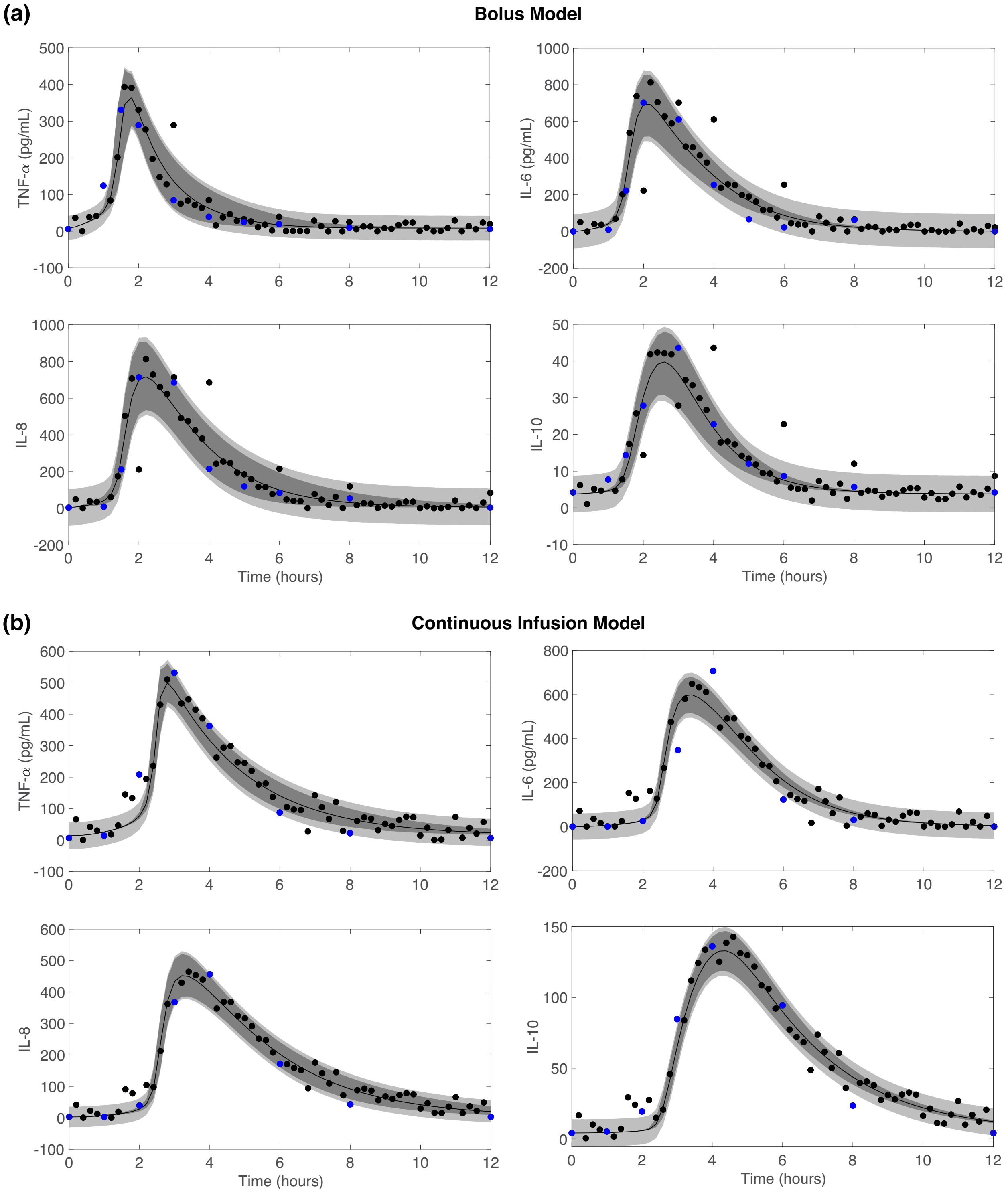

Figure S37: DRAM prediction (light gray) and credible (dark gray) intervals for the bolus (a) and continuous infusion (b) mean model response. Blue data points on (a) are from Janum et al. (2016) and black points are generated data. Blue data points on (b) are from Berg et al. (2012) and black points are generated data.

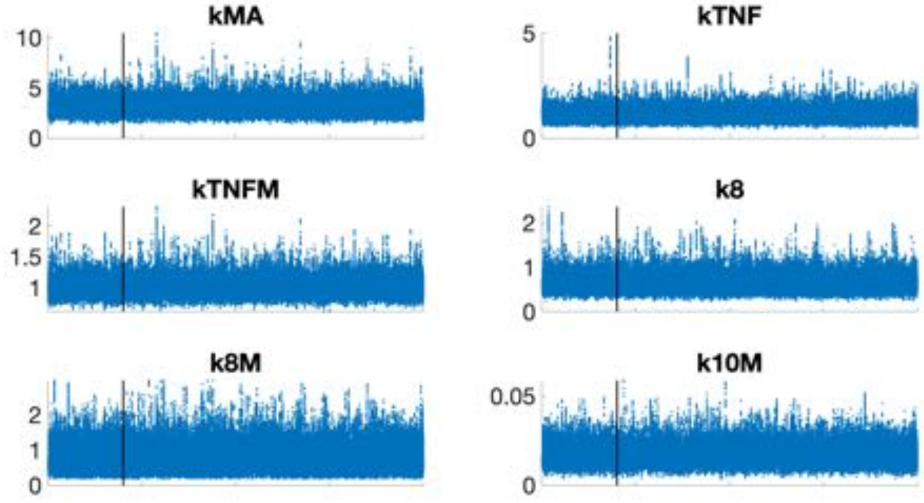

Figure S38: DRAM parameter chains for the optimized parameter set S_{Final} for the bolus model. 200,000 simulations were run with a burn-in period of 40,000. The black line represents the end of the burn-in period.

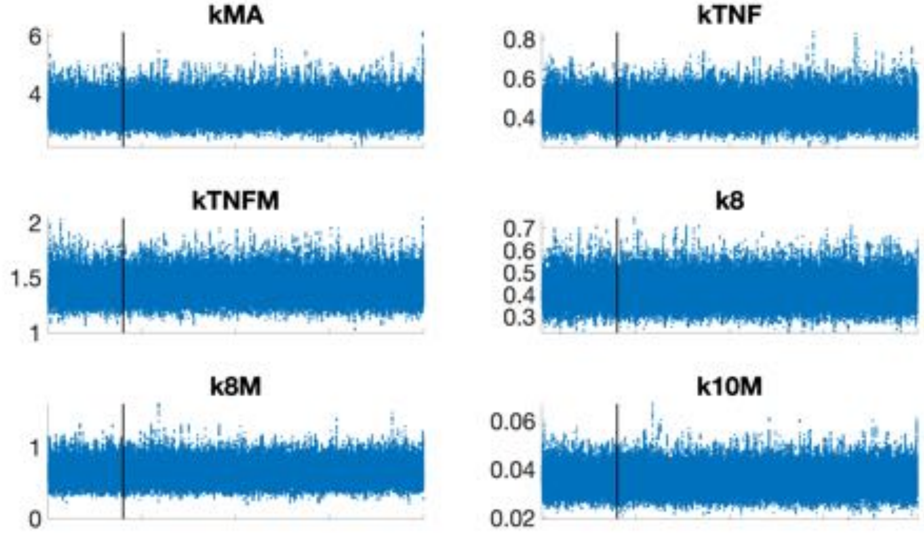

Figure S39: DRAM parameter chains for the optimized parameter set S_{Final} for the continuous infusion model. 200,000 simulations were run with a burn-in period of 40,000. The black line represents the end of the burn-in period.

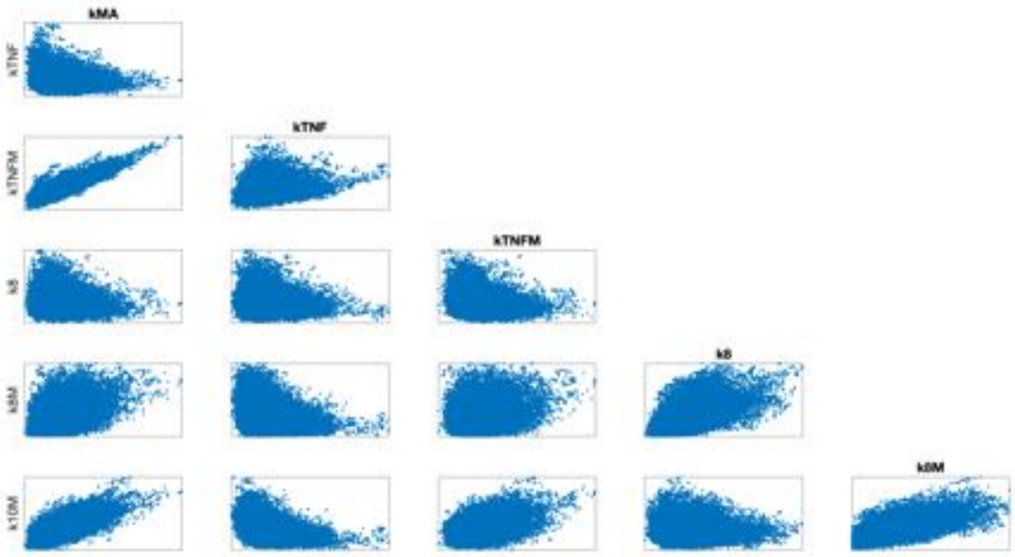

Figure S40: DRAM parameter correlations for the optimized parameter set S_{Final} for the bolus model. 200,000 simulations were run with a burn-in period of 40,000.

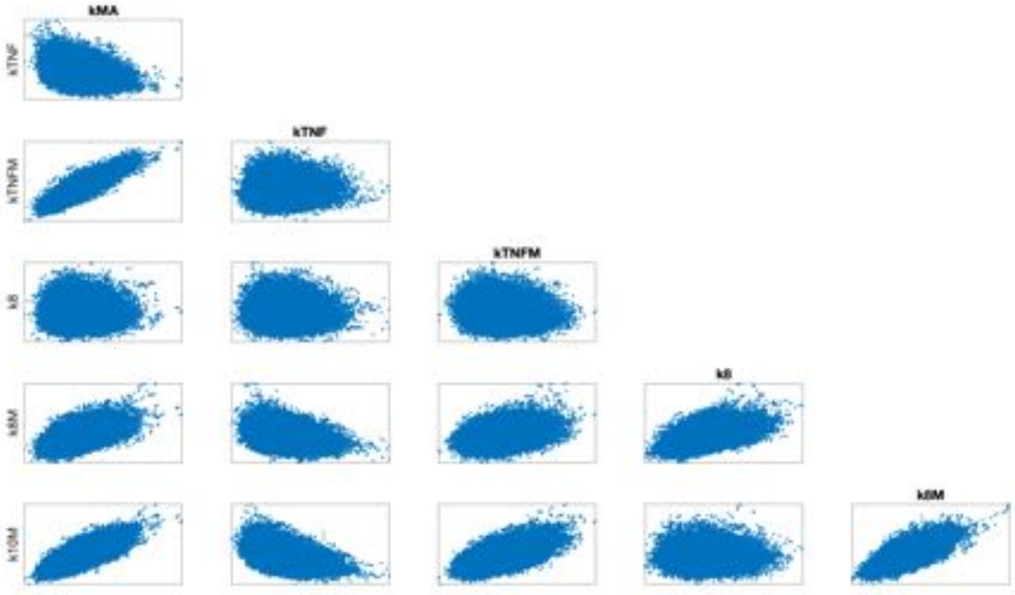

Figure S41: DRAM parameter correlations for the optimized parameter set S_{Final} for the continuous infusion model. 200,000 simulations were run with a burn-in period of 40,000.

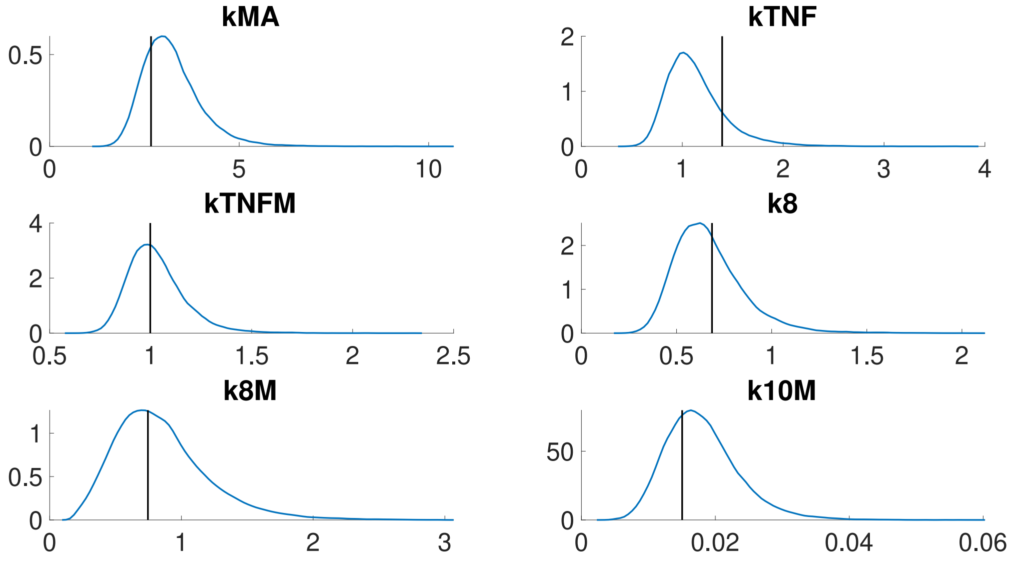

Figure S42: DRAM parameter distributions for the optimized parameter set S_{Final} for the bolus model. 200,000 simulations were run with a burn-in period of 40,000. The black line represents the bolus optimized parameter values reported in Table 4 in the main manuscript.

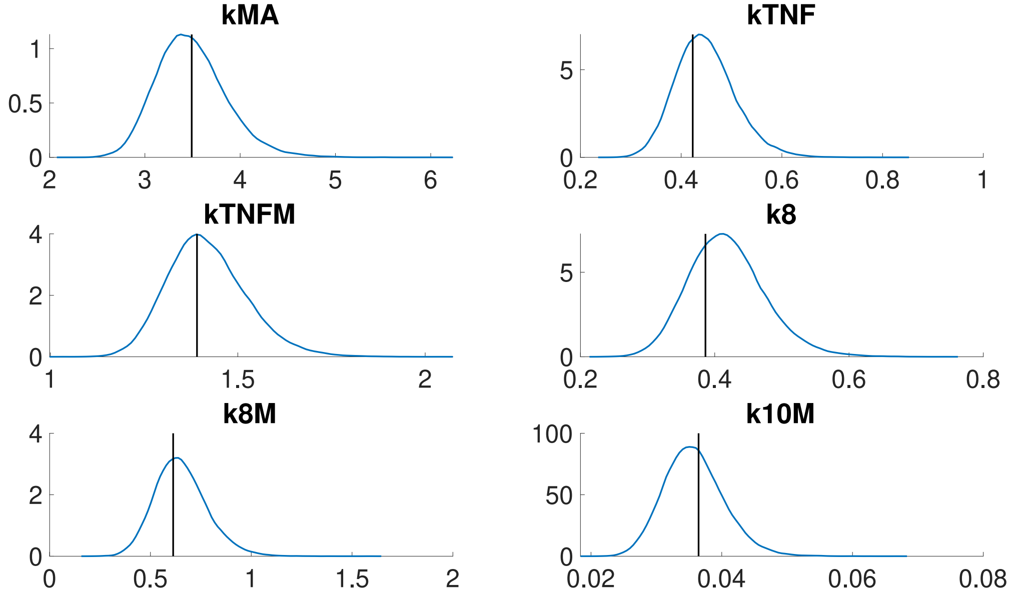

Figure S43: DRAM parameter distributions for the optimized parameter set S_{Final} for the continuous infusion model. 200,000 simulations were run with a burn-in period of 40,000. The black line represents the continuous infusion optimized parameter values reported in Table 4 in the main manuscript.

References

Banks, H., Davidian, M., Samuels, J., & Sutton, K. (2009). An inverse problem statistical methodology summary. In *Mathematical and Statistical Estimation Approaches in Epidemiology*, pages 249–302. Springer, Dordrecht, Netherlands.

Berg, R., Plovsing, R., Ronit, A., Bailey, D., Holstein-Rathlou, N., & Møller, K. (2012). Disassociation of static and dynamic cerebral autoregulatory performance in healthy volunteers after lipopolysaccharide infusion and in patients with sepsis. *Am J Physiol*, 303,R1127–R1135.

Burnham, K. & Anderson, D. (2002). *Model selection and multi-model inference: a practical information-theoretic approach*. Springer-Verlag.

Chihara, L. M. & Hesterberg, T. C. (2011). *Mathematical Statistics with Resampling and R*. John Wiley & Sons, Hoboken, New Jersey.

Dodge, Y. (2008). *The concise encyclopedia of statistics*. Springer Science & Business Media.

Fornacon-Wood, I., Mistry, H., Johnson-Hart, C., Faivre-Finn, C., O'Connor, J. P., & Price, G. J. (2022). Understanding the differences between bayesian and frequentist statistics. *International journal of radiation oncology, biology, physics*, 112(5),1076–1082.

Haario, H., Laine, M., Mira, A., & Saksman, E. (2006). Dram: efficient adaptive mcmc. *Statistics and computing*, 16,339–354.

Janum, S., Nielsen, S., Werner, M., Mehlsen, J., Kehlet, H., & Møller, K. (2016). Pain perception in healthy volunteers: Effect of repeated exposure to experimental systemic inflammation. *Innate Immun*, 22,546–556.

Lye, A., Cicirello, A., & Patelli, E. (2021). Sampling methods for solving bayesian model updating problems: A tutorial. *Mechanical Systems and Signal Processing*, 159,107760.

Miao, H., Xia, X., Perelson, A. S., & Wu, H. (2011). On identifiability of nonlinear ode models and applications in viral dynamics. *SIAM Rev*, 53,3–39.

Olufsen, M. & Ottesen, T. (2013). A practical approach to parameter estimation applied to model predicting heart rate regulation. *J Math Biol*, 67,39–68.

Pope, S. R., Ellwein, L. M., Zapata, C. L., Novak, V., Kelley, C. T., & Olufsen, M. S. (2009). Estimation and identification of parameters in a lumped cerebrovascular model. *Math Biosci Eng*, 6(1),93–115.

Schwarz, G. (1978). Estimating the dimension of a model. *Ann Stat*, pages 461–464.

Seber, G. & Wild, C. (2003). *Nonlinear regression*. Wiley-Interscience Paperback Series. Wiley, Hoboken, NJ.

Smith, R. (2013). *Uncertainty quantification: theory, implementation, and applications*. SIAM, Philadelphia, PA.

Windoloski, K., Bangsgaard, E., Dobreva, A., Ottesen, J., & Olufsen, M. (2023). A unified computational model for the human response to lipopolysaccharide-induced inflammation. In *Multiplicity of time scales in complex systems*, Mathematics Online First Collections. Springer Cham, Switzerland.
